# A bifunctional cellulase–xylanase of a new *Chryseobacterium* strain isolated from the dung of a straw‐fed cattle

**DOI:** 10.1111/1751-7915.13034

**Published:** 2017-12-04

**Authors:** Hao Tan, Renyun Miao, Tianhai Liu, Lufang Yang, Yumin Yang, Chunxiu Chen, Jianrong Lei, Yuhui Li, Jiabei He, Qun Sun, Weihong Peng, Bingcheng Gan, Zhongqian Huang

**Affiliations:** ^1^ National‐local Joint Engineering Laboratory of Breeding and Cultivation of Edible and Medicinal Fungi, Soil and Fertilizer Institute Sichuan Academy of Agricultural Sciences Chengdu China; ^2^ Scientific Observing and Experimental Station of Agro‐microbial Resource and Utilization in Southwest China Ministry of Agriculture Chengdu China; ^3^ College of Life Sciences Sichuan University Chengdu China

## Abstract

A new cellulolytic strain of *Chryseobacterium* genus was screened from the dung of a cattle fed with cereal straw. A putative cellulase gene (*cbGH5*) belonging to glycoside hydrolase family 5 subfamily 46 (GH5_46) was identified and cloned by degenerate PCR plus genome walking. The CbGH5 protein was overexpressed in *Pichia pastoris*, purified and characterized. It is the first bifunctional cellulase–xylanase reported in GH5_46 as well as in *Chryseobacterium* genus. The enzyme showed an endoglucanase activity on carboxymethylcellulose of 3237 μmol min^−1^ mg^−1^ at pH 9, 90 °C and a xylanase activity on birchwood xylan of 1793 μmol min^−1^ mg^−1^ at pH 8, 90 °C. The activity level and thermophilicity are in the front rank of all the known cellulases and xylanases. Core hydrophobicity had a positive effect on the thermophilicity of this enzyme. When similar quantity of enzymatic activity units was applied on the straws of wheat, rice, corn and oilseed rape, CbGH5 could obtain 3.5–5.0× glucose and 1.2–1.8× xylose than a mixed commercial cellulase plus xylanase of Novozymes. When applied on spent mushroom substrates made from the four straws, CbGH5 could obtain 9.2–15.7× glucose and 3.5–4.3× xylose than the mixed Novozymes cellulase+xylanase. The results suggest that CbGH5 could be a promising candidate for industrial lignocellulosic biomass conversion.

## Introduction

Crop straw is a major agricultural waste as well as an abundant resource of lignocellulosic biomass. The global production of straw is estimated to reach at least 1500 million tons per year, in which China contributes over 700 million tons (Sun, 2010). Inadequate recycling and treatment of crop straw such as burning may cause severe environmental problems, for instance, particulate matter (PM) 2.5 pollution in the air (Libo *et al*., 2016). To utilize crop straw in ecological friendly ways, there is a growing need to convert the polysaccharides in straw to saccharides (Gupta and Verma, 2015), by physiochemical treatments and enzymatic degradation. In another way, crop straw is a main substrate in oyster mushroom cultivation (*Pleurotus ostreastus*) (Zhang *et al*., 2002). After oyster mushroom harvesting, the residual cultivation substrate is called spent mushroom substrate, which was estimated to reach 50 million tons per year in China (Wu *et al*., 2013). Spent mushroom substrate is also rich in lignocellulosic biomass, which could be converted to saccharides and biofertilizers (Zhu *et al*., 2013).

Saccharification effects on both crop straw and spent mushroom substrate are greatly influenced by catalytic performance of cellulase during enzymatic treatments, which play a crucial role in lignocellulose degradation (Jiang *et al*., 2016). As high temperature is beneficial for converting lignocellulosic biomass to saccharides (Wang *et al*., 2015; Shirkavand *et al*., 2016), cellulase with high activity and stability at high‐temperature is favoured (Yeoman *et al*., 2010). Most of the currently known thermophilic cellulases are optimum at 50–70 °C (Bischoff *et al*., 2006; Assareh *et al*., 2012; DeCastro *et al*., 2016), while hyperthermophilic cellulases with optimum temperature around 80–90 °C are not very common (DeCastro *et al*., 2016).

In addition, the hydrolytic performance of cellulase could be enhanced by collaboration with hemicellulase (xylanase, in particular) (Jia *et al*., 2016), since deconstruction of hemicellulose layers between cellulose microfibers further improves the accessibility of cellulose to hydrolytic enzymes(Meng and Ragauskas, 2014). Accordingly, bifunctional enzymes possessing cellulase and xylanase activities could be more effective. In previous studies, bifunctional cellulase–xylanase enzymes with optimum temperatures around 50–65 °C were found in rumen microbial communities (Chang *et al*., 2011; Rashamuse *et al*., 2013; Cheng *et al*., 2015), which are always rich sources of cellulolytic microbes and biomass‐degrading genes (Hess *et al*., 2011).

In this study, a microbial community of bovine dung was screened to find a thermophilic cellulase with high activity and preferably with auxiliary xylanase activity, which is expected to have improved saccharification effects on straw and spent mushroom substrate.

## Results

### Isolation of *Chryseobacterium* sp. HT1

Thermophilic cellulolytic microbes in the dung of the straw‐fed cattle were enriched in a liquid medium supplemented with wheat straw powder as sole carbon source at 55 °C and further screened on agar plates with carboxymethylcellulose (CMC) as sole carbon source. Eighty‐seven of 4341 colonies generated clearing zone on the plates, indicating that the isolates are probably cellulase producers. The one with the largest clearing zone was picked up. The microbe was a bacterium, forming chrome‐yellow, round, glossy and smooth‐edged colonies (Fig. S1).

**Figure 1:**
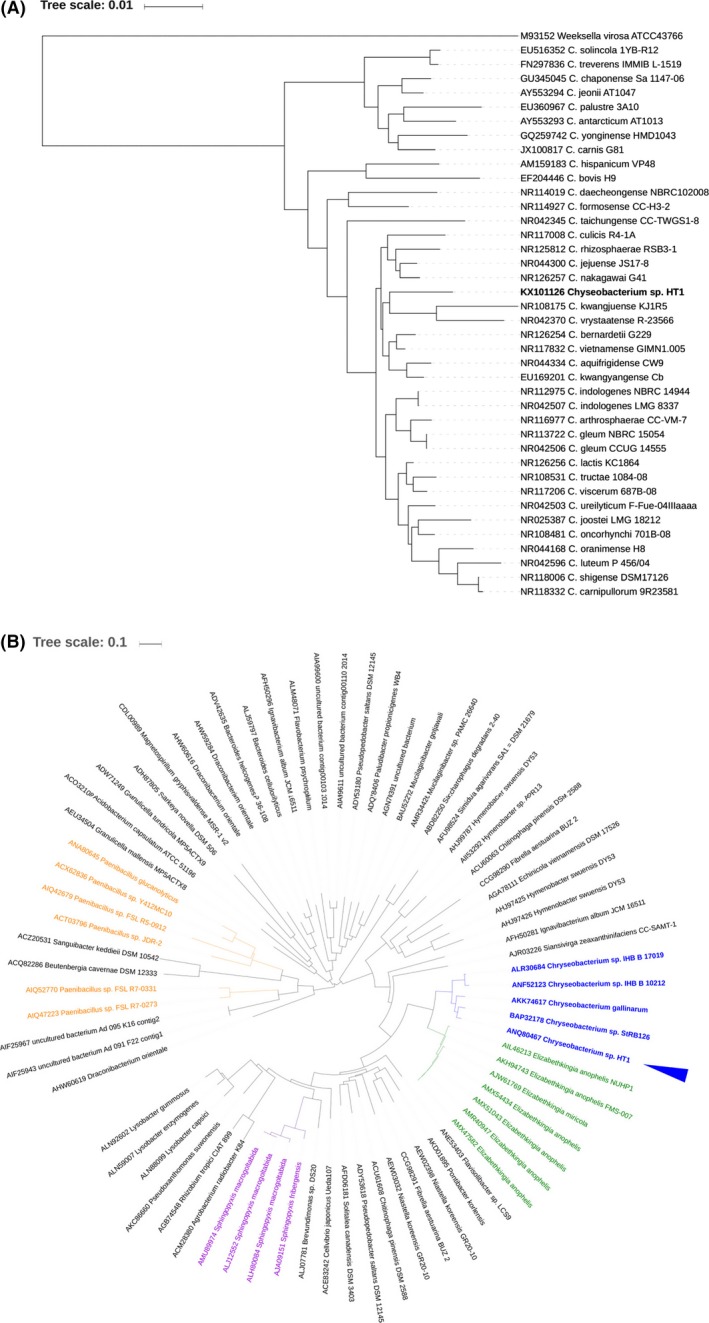
A. Phylogenetic analysis of 16S rRNA gene sequences of *Chryseobacterium* sp. HT1 with 45 reference strains of *Chryseobacterium* genus. B. Phylogenetic analysis of AA sequences of CbGH5 with 70 putative proteins of GH5_46 from the CAZy database.

16S rRNA gene sequencing (GenBank accession number: KX101126) revealed that the bacterium might belong to *Chryseobacterium* genus. A phylogenetic analysis of 16S rRNA gene sequences showed that the closest strains (96.6% similar, by BLASTn) are *C. kwangjuense* KJ1R5 and *C. vrystaatense* R‐23566 (Fig. 1A). It means that the bacterium could be a new *Chryseobacterium* strain. The strain was named *Chryseobacterium* sp. HT1.

### Identification of cbGH5, a putative cellulase gene

**Figure 2:**
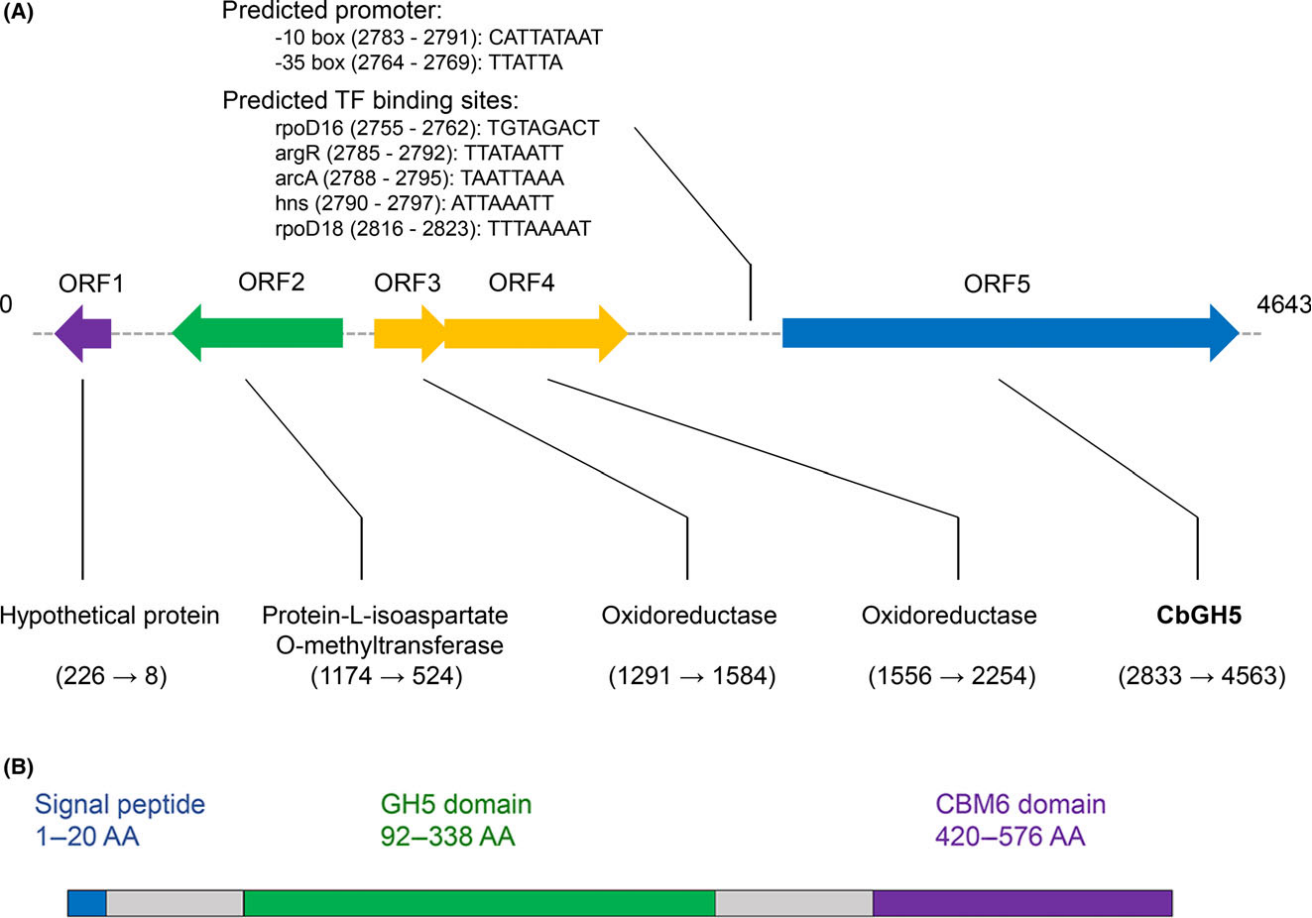
A. Schematic representations of predicted *cbGH5* and upstream ORFs. B. Three major modules of the CbGH5 peptide.

To amplify putative cellulase gene in *Chryseobacterium* sp. HT1, degenerate primers were designed using 27 putative cellulase genes of *Chryseobacterium* microbes. A 790 bp fragment was obtained and then assembled with its flanking regions retrieved by genome walking, which finally generated a 4643 bp fragment (GenBank accession number: KX101127). Five open reading frames (ORFs) were identified in this fragment (Fig. 2A). The ORFs 1–4 were predicted to encode two oxidoreductases, a protein‐L‐isoaspartate *O*‐methyltransferase and an unknown protein respectively. The ORF 5 (1731 bp) was predicted to encode a 576 amino acids (AA) product (GenBank accession number: ANQ80467), 89% similar (by blastp) to a glycoside hydrolase family 5 of *Chryseobacterium* sp. StRB126 (GenBank accession number: BAP32178), which is most likely a cellulase gene among all the five ORFs. The gene was named *cbGH5*. The two oxidoreductases were predicted to form an operon, while the putative cellulase, the protein‐L‐isoaspartate *O*‐methyltransferase and the unknown protein were predicted as individual transcriptional units. A −10 box and a −35 box were predicted to locate upstream the *cbGH5* gene, with putative binding sites of transcriptional factors rpoD16, argR, arcA, hns and rpoD18 (Fig. 2A). This region might be the promoter of *cbGH5*.

After submitting to GenBank, the AA sequence of CbGH5 was automatically included by the CAZy Database website (http://www.cazy.org/GH5_46_bacteria.html) and classified as a glycoside hydrolase (GH) family 5 subfamily 46 (GH5_46) protein by the integrated annotating tool of the database. A phylogenetic analysis of GH5_46 proteins showed that CbGH5 clustered together with four proteins derived from other *Chryseobacterium* microbes (Fig. 1B), suggesting that the AA sequence of CbGH5 homologues seems highly conserved in *Chryseobacterium* genus. Sequence alignment revealed that Glu215 and Glu312 of CbGH5 are conserved in these GH5_46 proteins (Fig. S2), which are very possibly the two AA residues are the key glutamate pair in the catalytic site of GH5 family proteins.

CbGH5 possesses a N‐terminal signal peptide in the 1–20 AA region (Fig. 2B), as predicted by SignalP 4.1 (Petersen *et al*., 2011). It means that CbGH5 might be a secreted protein in *Chryseobacterium* sp. HT1. The protein consists of a conserved domain of GH5 (length: 247 AA) near the N‐terminal and a carbohydrate‐binding module family 6 domain (CBM6, length: 157 AA) near the C‐terminal, connected by an 81 AA linker (Fig. 2B), as predicted by the online conserved domain identifier integrated in the NCBI blastp tool.

### Overexpression and purification of CbGH5

CbGH5 was overexpressed as a recombinant protein in *Pichia pastoris* GS115 (Table S1). Molecular weight (Mw) and homogeneity of the purified protein were initially checked by SDS‐PAGE (Fig. S3A) and further determined by MALDI‐TOF analysis (Fig. S3B). The Mw of de‐glycosylated CbGH5 shown by MALDI‐TOF is 65892.97 Da, basically in agreement with the theoretical Mw 65895.23 Da, suggesting that CbGH5 is monomeric.

### Bifunctional cellulase–xylanase activities

**Figure 3:**
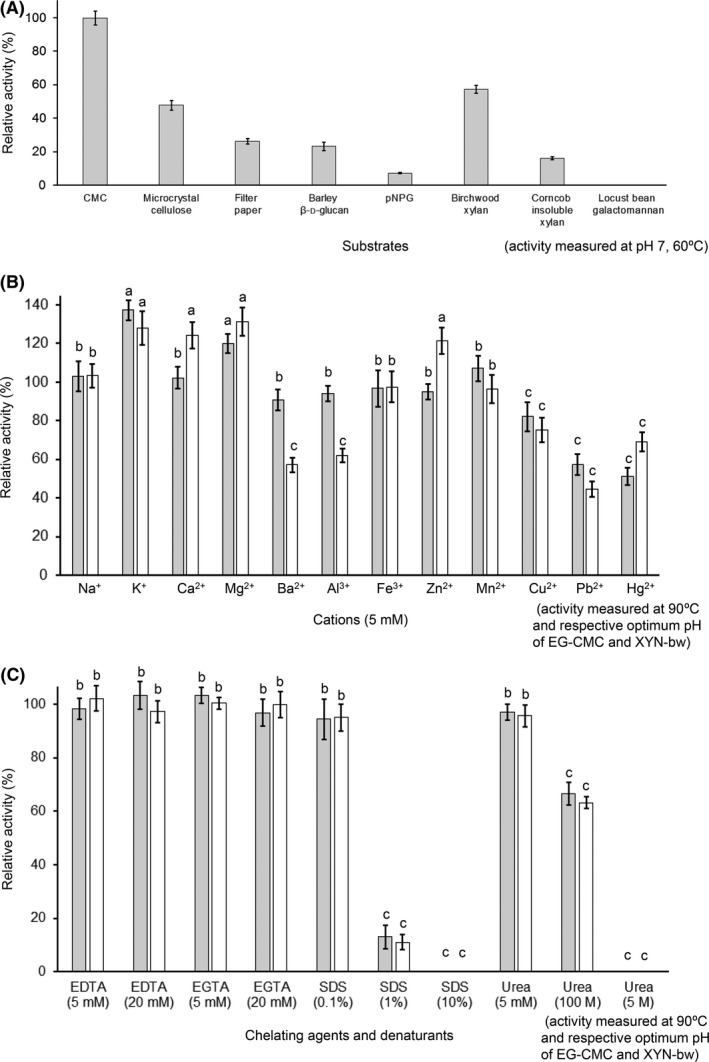
A. Substrate preference of CbGH5. B. Effects of cations on the EG‐CMC (grey bar) and XYN‐bw (white bar) activities. All the cations were supplemented at a final concentration of 5 mM. The anion for the chemicals used is Cl^−^. C. Effects of chelating agents and denaturants. The final concentrations of EDTA, EGTA, SDS and urea were given in parentheses. For figures B and C, the activity without adding any cation, chelating agent or denaturant is defined as 100%. (a) significantly higher than 100%, (b) no significant difference with 100%, (c) significantly lower than 100%.

To determine the substrate specificity of CbGH5, hydrolytic activity was initially tested on different substrates (Table S2) at pH 7, 60 °C. The results showed that CbGH5 had higher activities on CMC, birchwood xylan and microcrystalline cellulose, while the activities on filter paper, barley β‐D‐glucan, *p*‐nitrophenyl β‐D‐glucoside (*p*NPG) and corncob insoluble xylan were lower (Fig. 3A). It showed no activity on mannan. The activities on insoluble cellulose and xylan were obviously lower than on soluble cellulose and xylan. The results mean that the enzyme possesses both endo‐cellulase and xylanase activities. Thus, the activities for CMC and birchwood were chosen for representing the cellulase and xylanase activities of CbGH5, respectively, which were abbreviated as EG‐CMC and XYN‐bw.

### pH and temperature profiles

**Figure 4:**
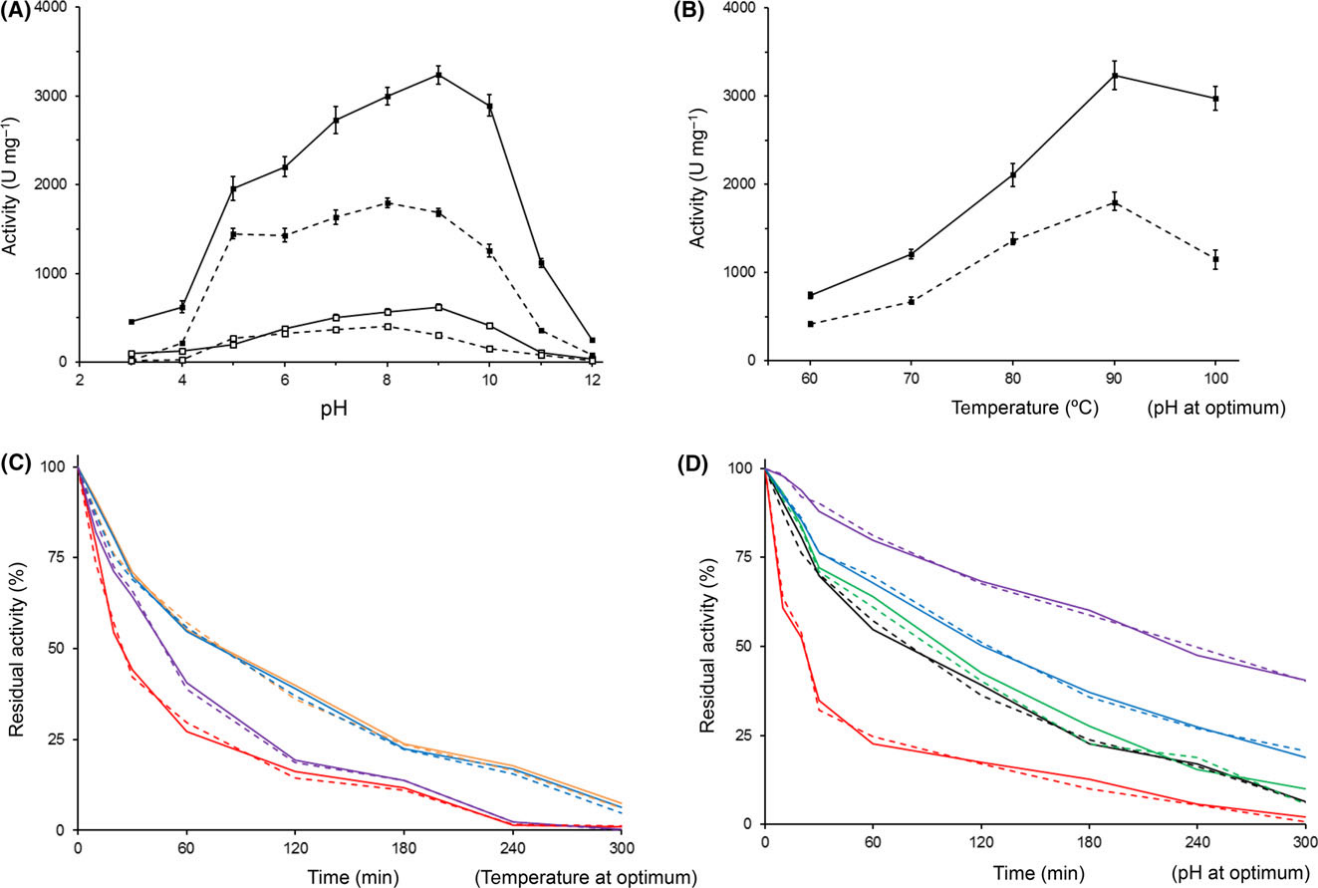
A. pH profiles of the EG‐CMC (solid line) and XYN‐bw (dash line) activities of CbGH5 at 60 °C (empty square) and 90 °C (solid square). B. Temperature profiles of the EG‐CMC and XYN‐bw determined at their respective optimum pH. C. Stability of the EG‐CMC and XYN‐bw activities at pH 4 (red), 8 (orange), 9 (blue) and 11 (purple). D. Stability of the EG‐CMC and XYN‐bw activities at 60 °C (purple), 70 °C (blue), 80 °C (green), 90 °C (black) and 100 °C (red). Standard deviation bars were not shown in figures C and D, for clearer visualization.

pH and temperature profiles were determined for both the EG‐CMC and XYN‐bw activities. The pH profiles demonstrated similar trends at 60 and 90 °C (Fig. 4A). The EG‐CMC activity was highest at pH 9 and XYN‐bw at pH 8, suggesting that the enzyme is slightly alkaliphilic. Between pH 6 and 10, CbGH5 demonstrated more than 68% of the EG‐CMC activity and 70% of the XYN‐bw, suggesting moderate adaptability to pH. The EG‐CMC and XYN‐bw activities of CbGH5 had the same optimal temperature (90 °C). The EG‐CMC and XYN‐bw activities at 90 °C were approximately three times those of 70 °C (Fig. 4B), suggesting that CbGH5 is thermophilic. The highest EG‐CMC activity (3237 ± 104 U mg^−1^) was observed at pH 9, 90 °C while the highest XYN‐bw activity (1793 ± 55 U mg^−1^) was at pH 8, 90 °C.

### pH and temperature stability

The EG‐CMC and XYN‐bw activities of CbGH5 showed quite similar stability under all the conditions tested (Fig. 4C and D), as expected. First, the enzyme was incubated at different pH at 90 °C. The results showed that the enzyme was more stable at weak alkalic pH (8 and 9), less stable at strong alkalic pH (11) and was vulnerable to medium acidic pH (4) (Fig. 4C). In addition, the stability at pH 8 and 9 was close. Then it was incubated at different temperatures at pH 8 (for the XYN‐bw activity) or 9 (for EG‐CMC). The results showed that CbGH5 had deduced half‐lifetime (*t*_1/2_) over 225 min at 60 °C but could be quickly inactivated when boiled (100 °C). Between 70 and 90 °C, the enzyme showed a moderate stability. The EG‐CMC and XYN‐bw activities both had deduced *t*_1/2_ near 80 min under their respective optimum conditions (pH 9 or 8, 90 °C).

### Effects of cations, chelating agents and denaturants

The effects of metal ions (at 5 mM concentration) on the EG‐CMC and XYN‐bw activities were assessed (Fig. 3B). The results showed that K^+^ and Mg^2+^ significantly enhanced the EG‐CMC and XYN‐bw activities, while Na^+^ and Fe^3+^ had no obvious effect. Zn^2+^ could slightly lower the EG‐CMC activity, and Ca^2+^ showed almost no effect, while they both enhanced the XYN‐bw activity significantly. Ba^2+^ and Al^3+^ slightly lowered the EG‐CMC activity and significantly decreased the XYN‐bw activity. Heavy metal ions such as Cu^2+^, Pb^2+^ and Hg^2+^ all demonstrated significant negative effects, except for Mn^2+^ that slightly enhanced the EG‐CMC activity. The EG‐CMC and XYN‐bw activities of CbGH5 seemed not cation‐dependent, as 5 and 20 mM of EDTA or EGTA showed no significant influence on the activity level (Fig. 3C).

0.1% SDS and 5 mM of urea had no significant inhibition on the EG‐CMC and XYN‐bw activities. 1% SDS and 100 mM of urea showed obvious inhibition, while 10% SDS and 5 M of urea completely inactivated the enzyme (Fig. 3C). It suggests that CbGH5 could withstand tiny concentration of the denaturants but were not resistant to medium and high concentrations.

### Structural model

The structural model of CbGH5 was predicted by NovaFold (DNASTAR Inc., Madison, WI, USA). The AA sequence was threaded through two templates (Fig. S4): the GH5‐CBM6 arabinoxylan‐specific xylanase CtXyl5A of *Clostridium thermocellum* [PDB accession numbers: 2Y8K (Correia *et al*., 2011)] and the *exo*‐β‐1,3‐glucanase Exg from *Candida albicans* [PDB accession numbers: 1CZ1 (Cutfield *et al*., 1999)]. A synthetic structural model was then deduced.

**Figure 5:**
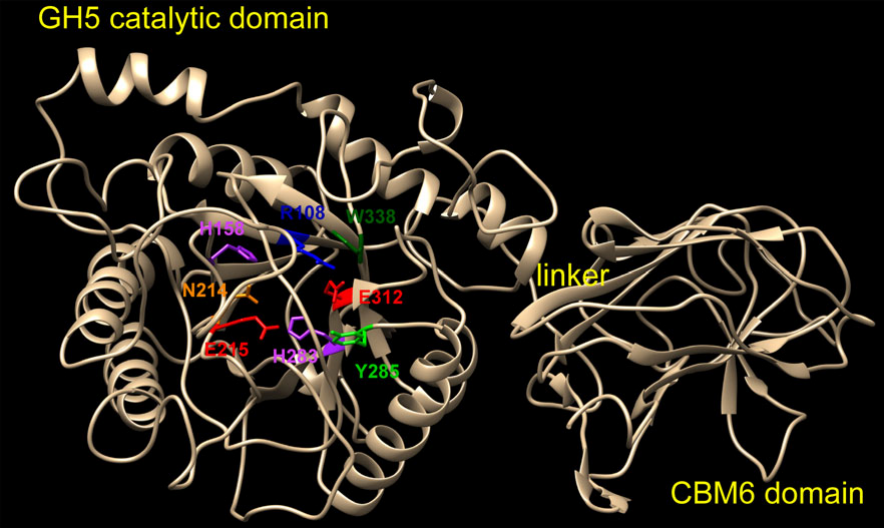
Homology model of CbGH5 showing the GH5 domain with (β/α)_8_ TIM‐barrel fold, the linker and the CBM6 domain. The two key glutamate residues in charge of the catalytic mechanism are highlighted in red. The conserved Asn, His, Arg, Tyr and Trp residues near the catalytic centre are in other colours.

The model consists of three major parts (Fig. 5): a catalytic domain of GH5, a linker and a CBM6 domain. No disulphide bridge was identified in the structural model. The GH5 domain has a typical (β/α)_8_ TIM‐barrel fold (Höcker *et al*., 2001). Eight parallel β‐strands form an inner tube, surrounded by an outer shell containing eight α‐helices. The Glu215 and Glu312 residues locate on the inner tube. Site mutagenesis converting either glutamate to alanine led to loss of the EG‐CMC and XYN‐bw activities (Table S4), indicating that two glutamates are indeed the catalytic residues as the acid/base donor and the nucleophile respectively (Yennamalli *et al*., 2011).

Disuphide bridge was not found in the structural model, suggesting that the thermophilicity of CbGH5 was not determined by the anchoring effect of disulphide bridge. About 59% of the AA in the eight parallel β‐strands are hydrophobic, while the eight α‐helices are more hydrophilic (47% of hydrophobic AA). As core hydrophobicity is a typical feature of thermophilic glycoside hydrolase (Yennamalli *et al*., 2011), it was hypothesized that the hydrophobicity of the β‐strand core might have an effect on the thermophilicity. To confirm this, a multiple‐site mutation protein CbGH5 (I107G, L109G, L153G, L155G, L157G, F257G, F280G, F282G) was overexpressed and purified with the same procedures for the wild‐type protein, using a mutant plasmid constructed by direct chemical synthesis. The overall skeleton of the mutant is not greatly altered in comparison with the wild‐type CbGH5 (Fig. S5). The mutant protein lowered the proportion of hydrophobic AA in the eight β‐strands from 59% to 39%. As a result, the optimum temperature for the EG‐CMC and XYN‐bw activities of the mutant protein was lowered by 20 °C (from 90 to 70 °C). It showed that the core hydrophobicity of CbGH5 has indeed a positive effect on the thermophilicity.

### Effects of CBM6

**Figure 6:**
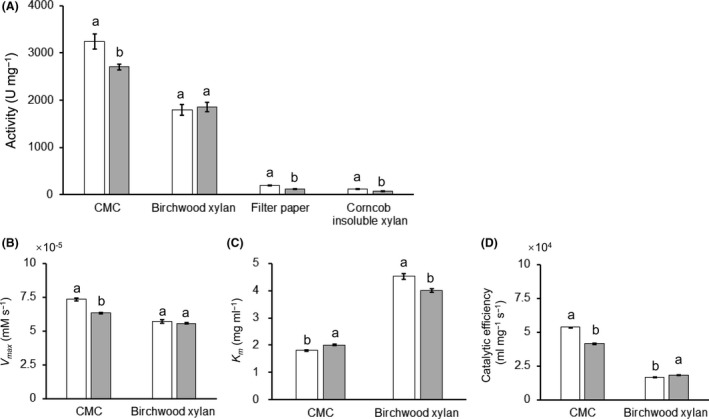
Influence of deleting the CBM6 domain of CbGH5 on the activity level (A), maximum reaction velocity (*V*_max_) (B), Michaelis constant (*K*_m_) (C) and catalytic efficiency (*k*_cat_ *K*_m_^−1^) (D). Significant difference is labelled by a and b. The values of the data bars in the figures are provided in the Supporting information (Table S5). Eadie‐Hofstee plots for determination of *K*_m_ and *V*_max_ are shown in supplementary figure (Fig. S7). CMC and filter paper were used as soluble and insoluble cellulose substrates, while birchwood xylan and corncob xylan were used as soluble and insoluble xylan substrates respectively. The activity levels and kinetic parameters were all measured under respective optimum pH and temperature conditions. For the insoluble substrates filter paper and corncob xylan, measuring kinetic parameters is not applicable, because insoluble substance is unable to form homogeneous solution, which is used for making a gradient of substrate concentration requested in measurement of kinetic parameters.

The function of the CBM6 domain of CbGH5 was tested. A mutant (CbGH5ΔCBM6) lacking the CBM6 was constructed, overexpressed and purified in the same way as CbGH5. The endoglucanase activity was tested on CMC (soluble) and filter paper (insoluble), while the xylanase activity was tested on birchwood xylan (soluble) and corncob xylan (insoluble). A comparison was made between the CBM6‐truncated mutant and the wild‐type (Fig. 6A–D).

When tested on soluble cellulose and xylan, deletion of the CBM6 significantly decreased the activity level for CMC but slightly influenced the activity level for birchwood xylan (Fig. 6A). The *V*_max_ for CMC was significantly decreased, while the change of the *V*_max_ for birchwood xylan was not obvious (Fig. 6B). The *K*_m_ for CMC was significantly raised while the *K*_m_ for birchwood xylan showed an opposite trend (Fig. 6C). It suggests that deletion of the CBM6 decreased the affinity of the enzyme for CMC but increased the affinity for birchwood xylan. The catalytic efficiency (*k*_cat_ *K*_m_^−1^) for CMC was significantly lowered, while the catalytic efficiency for xylan was significantly raised (Fig. 6D).

When tested on insoluble cellulose and xylan, both activity levels were greatly lowered (Fig. 6A). It suggests that the CBM6 domain is critical for the enzyme to hydrolyse insoluble substrates.

### Saccharification performance on straw and spent mushroom substrate

Saccharification performance was tested on straws of wheat, rice, corn and oilseed rape, as well as spent mushroom substrates made from the four straws respectively. A comparison was made between CbGH5 and the mixed NS50013 cellulase plus NS50014 xylanase of Novozymes, using similar quantity of enzymatic activity units to hydrolyse for 2 h near respective optimum pH and temperature conditions.

**Figure 7:**
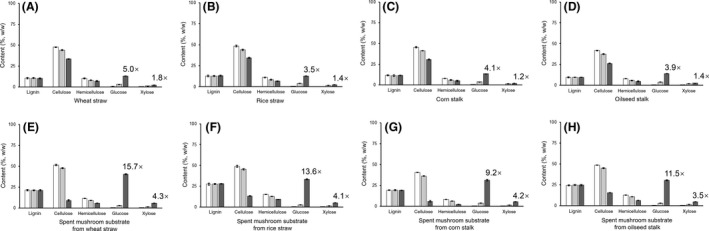
Saccharification performance of CbGH5 and the mixed commercial cellulase+xylanase of Novozymes. Contents of lignin, cellulose, hemicellulose, glucose and xylose were measured before and after enzymatic hydrolysis, on the straws of wheat (A), rice (B), corn (C) and oilseed rape (D), as well as spent mushroom substrates made from the straws of wheat (E), rice (F), corn (G) and oilseed rape (H). White bar: raw material before hydrolysis. Light grey bar: hydrolysed by the mixed Novozymes cellulase+xylanase. Dark grey bar: hydrolysed by CbGH5. The folds of glucose and xylose released by CbGH5 compared with the mixed Novozymes cellulase+xylanase are labelled in the figures. The hydrolysis reaction was carried out for 2 h. The pH and temperature conditions were 4.8, 45 °C for the mixed Novozymes cellulase plus xylanase, and 8.5, 90 °C for CbGH5 respectively. The values of the data bars in the figures are provided in the supplementary data (Table S6).

For both the straws and spent mushroom substrates, CbGH5 showed more effective conversion of cellulose and hemicellulose than the Novozymes cellulase+xylanase (Fig. 7, Table S6). For example, the Novozymes cellulase+xylanase released 2.44 mg of glucose and 1.22 mg of xylose from 1 g of wheat straw, while CbGH5 released 12.26 and 2.17 mg (Table S6), which means that CbGH5 obtained 5.0× glucose release and 1.8× xylose release (Fig. 7A). From 1 g of spent mushroom substrate made from wheat straw, the Novozymes cellulase+xylanase released 2.55 mg of glucose and 1.31 mg of xylose, while CbGH5 released 39.91 and 5.61 mg (Table S6), which means that CbGH5 obtained 15.7× glucose release and 4.3× xylose release (Fig. 7E). Approximately 78% of the cellulose and 49% of the hemicellulose present in the spent mushroom substrate made from wheat straw were converted to glucose and xylose by CbGH5. Similar results were also observed on the other three kinds of spent mushroom substrates. For all the four spent mushroom substrates, CbGH5 could hydrolyse over half of the cellulose content (Fig. 7E–H). The results suggest that CbGH5 could have a better performance in hydrolysing spent mushroom substrate than unutilized straw.

## Discussion

A novel bifunctional cellulase–xylanase CbGH5 was identified from a new *Chryseobacterium* strain (*Chryseobacterium* sp. HT1). It was isolated from the dung of a cattle which were fed with cereal straw as the main carbon source. Long‐term diet or feeding regime can indeed alter gut microbiota composition (Claesson *et al*., 2012; O'Donnell *et al*., 2013), and the straw feeding is expected to select for microbes with cellulolytic enzymes. Previous studies reported *Chryseobacterium* microbes isolated from diverse environments such as rhizosphere soil (Young *et al*., 2005), wastewater (Kämpfer *et al*., 2003) as well as endosymbiotic in chicken (Kämpfer *et al*., 2014) and human (Holmes *et al*., 2013). As a possible endosymbiont in bovine gut, *Chryseobacterium* sp. HT1 might help with degradation of cellulose in feed. The bacterium was isolated from a mesophilic sample but could grow under high‐temperature condition (55 °C) and possesses a thermophilic cellulase–xylanase. It is not surprising that thermophilic microbes and thermophilic enzymes could be found in mesophilic environments (Brigidi *et al*., 2003; Ariza *et al*., 2013; Tan *et al*., 2016a,b). To our knowledge, CbGH5 is the only carbohydrate‐active enzyme in *Chryseobacterium* genus that has been overexpressed, purified and characterized. As the AA sequence of CbGH5 homologues is highly conserved in *Chryseobacterium* genus, they might potentially be interesting objects for mining cellulase or bifunctional cellulase–xylanase candidates. Bifunctional cellulase–xylanase activities were previously reported in GH7 (Nakazawa *et al*., 2008; Karnaouri *et al*., 2014; Pellegrini *et al*., 2015), GH10 (Ding *et al*., 2008; Hess *et al*., 2011) and GH61 (Jagtap *et al*., 2014) as well as in GH5_2 (Ghatge *et al*., 2014), GH5_4 (Chang *et al*., 2011; Hess *et al*., 2011; Rashamuse *et al*., 2013; Cheng *et al*., 2015; Rattu *et al*., 2016) and GH5_25 (Yuan *et al*., 2015) (Table 1), while CbGH5 is the first example in GH5_46. To date, CbGH5 and a metagenome‐derived protein 421339_68070/TW‐2 (GenBank accession number: ADX05696) (Hess *et al*., 2011) are the only two glycoside hydrolases of GH5_46 that have been characterized.

**Table 1:** Summary of the bifunctional cellulase–xylanase enzymes characterized to date

Protein name/ID	Family	Source	GenBank accession number	References	Maximum activity level and catalytic efficiency (at optimum pH and temperature)	
					EG‐CMC	XYN‐bw
Cel5	GH5_2	*Hahella chejuensis* KCTC 2396	ABC30636	(Ghatge *et al*., 2014)	77.19 U mg^−1^, 1.63 × 10^−2^ ml mg^−1^ s^−1^ a (at pH 6.5, 65 °C)	9.19 U mg^−1^, 1.83 × 10^−3^ ml mg^−1^ s^−1^ (at pH 5.5, 55 °C)
rBhcell‐xyl	GH5_4	*Bacillus halodurans* C‐125	BAB04322	(Rattu *et al*., 2016)	16.88 U mg^−1^, 2.85 ml mg^−1^ s^−1^ a (at pH 6, 60 °C)	6.7 U mg^−1^, 10.95 ml mg^−1^ s^−1^ a (at pH 6, 60 °C)
Cel5A	GH5_4	Bovine rumen metagenome	AFO64636	(Rashamuse *et al*., 2013)	2.73 U ml^−1^, N/A (at pH 9, 65 °C)	N/A
Cel5B	GH5_4	Bovine rumen metagenome	AFO64637	(Rashamuse *et al*., 2013)	2.1 U ml^−1^, N/A (at pH 9, 65 °C)	N/A
558318_19410/MH‐20	GH5_4	Cow rumen metagenome	ADX05703	(Hess *et al*., 2011)	N/A	N/A
Cel28a	GH5_4	Goat rumen metagenome	AHW46443	(Cheng *et al*., 2015)	20.56 U mg^−1^ (at pH 5, 50 °C)	9.02 U mg^−1^ (at pH 5, 50 °C)
RuCelA	GH5_4	Yak rumen metagenome	ADK55024	(Chang *et al*., 2011)	54.3 U mg^−1^, N/A (at pH 5, 50 °C)	264.1 U mg^−1^, N/A (at pH 7, 65 °C)
CtCel5E	GH5_25	*Ruminiclostridium thermocellum* ATCC 27405	ABN52701	(Yuan *et al*., 2015)	736.2 U mg^−1 ^g, 12.4 ml mg^−1^ s^−1^ a (at pH 5, 50 °C)	255.8 U mg^−1^, 3.2 ml mg^−1^ s^−1^ a (at pH 6, 60 °C)
CbGH5	GH5_46	*Chryseobacterium* sp. HT1	ANQ80467	This study	3237 U mg^−1^, 1.11 × 10^5^ ml mg^−1^ s^−1^ (at pH 9, 90 °C)	1793 U mg^−1^, 1.17 × 10^4^ ml mg^−1^ s^−1^ (at pH 9, 90 °C)
MtEG7a	GH7	*Myceliophthora thermophile* ATCC 42464	XM_003663393	(Karnaouri *et al*., 2014)	177 U mg^−1^, 0.314 ml mg^−1 ^s^−1^ a (at pH 5, 60 °C)	4 U mg^−1^, N/A (at pH 5, 60 °C)
Cel7B	GH7	*Trichoderma reesei* QM9414	AAA34212	(Nakazawa *et al*., 2008)	65 U mg^−1^, N/A (at pH 3, 50 °C)	3.9 U mg^−1^ a, N/A (at pH 3, 50 °C)
ThCel7B	GH7	*Trichoderma harzianum* IOC‐3844	N/A	(Pellegrini *et al*., 2015)	26 U mg^−1^, N/A (at pH 3, 55 °C)	12 U mg^−1^, N/A (at pH 3, 55 °C)
EgXA	GH10	*Ampullaria crossean*	AAP31839	(Ding *et al*., 2008)	7.5 U mg^−1^, N/A (at pH 5, 40 °C)	391.8 U mg^−1^, N/A (at pH 6, 45 °C)
3811766_163670/TW‐8	GH10	Cow rumen metagenome	ADX05679	(Hess *et al*., 2011)	N/A	N/A
3663344_18020/TW‐23	GH10	Cow rumen metagenome	ADX05674	(Hess *et al*., 2011)	N/A	N/A
3789863_192950/TW‐24	GH10	Cow rumen metagenome	ADX05745	(Hess *et al*., 2011)	N/A	N/A
AgEG	GH61	*Armillaria gemina* KJS114	N/A	(Jagtap *et al*., 2014)	1160 U mg^−1^, 3590 ml mg^−1^ s^−1^ (at pH 5, 60 °C)	1392 U mg^−1^ a, N/A (at pH 5, 60 °C)

N/A not available since the authors did not measure the parameters.

**a.** Value is calculated or deduced from original data given in the article.

Bioinformatic analyses predicted two oxidoreductases, a protein‐L‐isoaspartate *O*‐methyltransferase and an unknown protein upstream *cbGH5*. It is unlikely that *cbGH5* forms any operon with the upstream genes. Promoter prediction suggests that *cbGH5* expression might receive regulation from RNA polymerase sigma‐70 factor (Shimada *et al*., 2014). The overexpressed and purified CbGH5 showed maximum EG‐CMC and XYN‐bw activities at pH 9 and 8, respectively, which are both slightly alkalic. It differs from a number of cellulases and xylanases, whose optimum pH is usually acidic (Nakazawa *et al*., 2008; Ghatge *et al*., 2014; Jagtap *et al*., 2014; Karnaouri *et al*., 2014; Lafond *et al*., 2014; Wang *et al*., 2014; Cheng *et al*., 2015; Pellegrini *et al*., 2015). The enzyme is more stable at alkalic pH than acidic. It means that the enzyme might be a promising candidate for catalysis in alkalic environments. As alkaline pretreatment could be carried out under milder conditions than acid pretreatment (Kim *et al*., 2016) and is also more efficient than acid pretreatment in lignin removal (Wu *et al*., 2013), alkalic pH is more favoured than acidic in industrial (Meng and Ragauskas, 2014; Pollegioni *et al*., 2015). The enzyme could be applied after the alkaline pretreatment while minimal pH adjustment is needed.

The optimum temperature for both EG‐CMC and XYN‐bw activities is 90 °C, which means that CbGH5 is more thermophilic than many known thermostable/thermophilic cellulases and xylanases (Jang and Chen, 2003; Choi *et al*., 2006; Le and Wang, 2014; Li *et al*., 2014; Qiao *et al*., 2014) and is comparable with the endoglucanase from *Talaromyces emersonii* CBS394.64 (Wang *et al*., 2014). CbGH5 was more stable at 60 °C than 100 °C, which means that the thermostability can be further improved by engineering or immobilization for higher robustness in industrial application. The activity levels of EG‐CMC and XYN‐bw are higher than many known cellulases and xylanases. To date, only a few cellulase or xylanase possess high activity level comparable with CbGH5 (Borges *et al*., 2014; Lafond *et al*., 2014; Liu *et al*., 2015). Altogether, these characteristics make CbGH5 advantageous in cellulose and xylan catalysis.

The predicted structural model of CbGH5 contains no disulphide bridge. The number of disulphide bridge in many of the known thermophilic glycoside hydrolases is between 0 and 2 (Yennamalli *et al*., 2011). Having zero disulphide bridge is very common, for instance, the thermophilic CelC [PDB accession number: 1CEC (Dominguez *et al*., 1995)], CelCCA [PDB accession number: 1EDG (Ducros *et al*., 1995)] and Cel44A [PDB accession number: 2E4T (Kitago *et al*., 2007)] of *Ruminiclostridium cellulolyticum*, as well as the metagenome‐derived bifunctional glucanase–xylanase CelM2 [PDB accession number: 3II1 (Nam *et al*., 2010)]. The thermophilicity of these enzymes is not determined by the stabilizing effect of cysteine–cysteine link but is more likely by other conformational characteristics. In this study, we proved that the thermophilicity of CbGH5 is granted by core hydrophobicity. Hydrophobic interaction helps with forming the inner core of enzyme, which could thus enhance the stability and catalytic performance of enzyme at high‐temperature.

The CbGH5 mutant without CBM6 could retain both the EG‐CMC and XYN‐bw activities, suggesting that the bifunctional catalysis seems not mainly contributed by the CBM6. While very few bifunctional glycoside hydrolase is caused by two different GH catalytic domains in one enzyme [the 798985_48230/TW‐11 protein (Hess *et al*., 2011)] or two different binding clefts with broad substrate specificity in one CBM domain (Henshaw *et al*., 2004), the catalytic activity on multiple substrates in most of the known bifunctional or multifunctional glycoside hydrolases seems contributed mainly by the GH catalytic domain. It is consistent with the phenomenon that the EG‐CMC and XYN‐bw activities of CbGH5 showed similar pH and temperature stability.

The EG‐CMC and XYN‐bw activities of CbGH5 are not cation‐dependent, different from a few Ca^2+^‐dependent cellulases and xylanases (Yazawa *et al*., 2011; Zhang *et al*., 2015). The enzyme received no negative effect from cations of macronutrients such as Na^+^, K^+^, Ca^2+^ and Mg^2+^ but could be severely inhibited by heavy metal ions, suggesting that CbGH5 could be suitable for usual lignocellulosic treatments but should avoid samples rich in heavy metal such as industrial wastewater.

For both CbGH5 and the mixed Novozymes cellulase+xylanase, spent mushroom substrates could obtain much higher release of glucose than straws. A possible reason might be the growth of oyster mushroom mycelia had an impact on the microstructure of straw material in the mushroom substrate, which might facilitate the catalysis of hydrolytic enzymes (Meng and Ragauskas, 2014). The extending and penetrating of mycelia led to collapse and deconstruction of the vascular and cell wall structures in straw (Shirkavand *et al*., 2016). The large quantity of peroxidases secreted by oyster mushroom mycelia (Elisashvili *et al*., 2008) could effectively remove the lignin envelop of lignocellulosic complex (Taniguchi *et al*., 2005) and further improve cellulose accessibility to enzymes. Using similar amount of enzymatic activity units, CbGH5 could release higher amount of glucose and xylose than the mixed Novozymes cellulase+xylanase. The advantage would be greater if using the same weight of enzyme protein. It means that CbGH5 has a better performance in the catalytic reactions.

In conclusion, this study describes the identification and biochemical characterization of the first GH5_46 enzyme with bifunctional cellulase and xylanase activities, which is encoded by a new *Chryseobacterium* strain. The bifunctional enzyme possesses preferable characteristics: high activity level and catalytic efficiency, high thermophilicity, and the advantage in saccharification of straw and spent mushroom substrate.

## Experimental procedures

### Screening of cellulolytic candidates

Cellulolytic microbes were screened from the dung of an 8‐year‐old cattle. The cattle were raised by a peasant family for ploughing, in Tanguanyao Village group 1 (29.86N, 103.88E), Baiguo Town, Qingshen county, Meishan City, Sichuan Province, China. The cattle were fed with straw of wheat, rice and corn as the main carbon source for long‐term (about 4 years). Fresh dung was collected by hanging a sterile bag behind the cattle, on 03/08/2014, delivered in an icebox at approximate 4 °C to the laboratory and immediately processed for the next step.

Thermophilic microbes with lignocellulosic utilizing ability were preliminarily enriched before screening. 1 g of the fresh dung was added into a 2000 ml flask containing 400 ml of a selective medium modified from the recipe described by Li *et al*. (2011). The medium contained 1% w v^−1^ of wheat straw powder (passed through a 40 mesh sieve), 0.05% w v^−1^ of yeast extract, 1.0 g l^−1^ K_2_HPO_4_, 1.0 g l^−1^ (NH_4_)_2_SO_4_, 1.0 g l^−1^ NaNO_3_, 0.5 g l^−1^ MgSO_4_·7H_2_O, 0.01 g l^−1^ FeSO_4_·7H_2_O, 0.5 g l^−1^ KCl, at pH 7.0. The culture was grown with 180 rpm shaking and then re‐inoculated into fresh selective medium every 48 h, at a volume ratio of 1:100. The incubation temperature was 37 °C for the first 48 h, and then raised by 2 °C every 48 h. After 2 weeks, the incubation temperature reached 55 °C and was kept for another 5 days.

The culture was then spread on carboxymethylcellulose (CMC) plates with 1% Congo red, as described by Hess *et al*. (2011). The colony with the largest clearing zone was considered as the strongest cellulase producer and was picked up for further study.

### Extraction of bacterial genomic DNA

*Chryseobacterium* sp. HT1 was grown in LB medium at 37°C for 15 h with 200 rpm shaking. Genomic DNA of the cellulase producer microbe was extracted with an EZup Column Bacteria Genomic DNA Purification Kit (Sangon Biotech Inc., Shanghai, China) using the procedures instructed by the manufacturer.

### Sequencing of 16S rRNA gene

16S rRNA gene was amplified with bacterial universal primers 27f and 1492r (Lane, 1991) and sequenced by Sangon Biotech Inc.

### Retrieving the putative cellulase gene with flanking sequences

The putative cellulase gene of *Chryseobacterium* sp. HT1 was retrieved by degenerate PCR. Twenty‐seven putative cellulase genes of *Chryseobacterium* were randomly selected from the NCBI nucleotide database. The nucleotides were aligned using MAFFT (Katoh and Toh, 2008). A pair of degenerate primers (CL‐F and CL‐R, Table S3) was designed based on conserved sequences (Fig. S6). PCR amplification used a 50 μl reaction system containing 45 μl of Platinum PCR SuperMix High Fidelity (Invitrogen, Carlsbad, CA, USA), 500 nM of each primer and 1 μg of the genomic DNA of *Chryseobacterium* sp. HT1. The PCR programme was set as: 95 °C initial denaturation for 2 min, [95 °C denaturation for 30 s, 52 °C annealing for 30 s, 68 °C extension for 1 min] × 30 cycles, 68 °C final extension for 2 min. The amplified fragment was cloned into a pCR2.1‐TOPO vector with a TOPO TA‐Cloning Kit (Invitrogen) using the procedures instructed by the manufacturer. The cloned fragment was sequenced by Sangon Biotech Inc.

To obtain the flanking regions of the amplified fragment, a genome walking approach was adopted, using A TaKaRa Genome Walking Kit (TaKaRa, Kusatsu, Shiga, Japan). All steps followed the instructions provided by the manufacturer. Three pairs of inner primers (Table S3) were designed and used for the genome walking. After the full length of the fragment was assembled, it was verified by sequencing again (by Sangon Biotech Inc.).

### Prediction of gene, operon, promoter and signal peptide

Putative genes and operons in the 4643 bp fragment were predicted using FGENESB (Solovyev and Salamov, 2011). Putative promoter was predicted using BPROM (Solovyev and Salamov, 2011). Putative signal peptide was detected using SignalP (Petersen *et al*., 2011).

### Phylogenetic analysis

The 16S rRNA gene sequence of *Chryseobacterium* sp. HT1 (GenBank accession number: KX101126) was clustered with 45 strains in *Chryseobacterium* genus as references. The 45 strains were all of the *Chryseobacterium* microbes with 16S rRNA gene available online, updated to 02/March/2016. *Weeksella virosa* ATCC43766 was used as an outer‐group (Holmes *et al*., 2013). Sequence alignment and neighbour‐joining tree construction were carried out using the procedures as previously described (Holmes *et al*., 2013).

The deduced AA sequence of CbGH5 was clustered with 70 putative proteins of GH family 5 subfamily 46 (GH5_46) obtained from the carbohydrate‐active enzymes (CAZy) Database website (http://www.cazy.org/GH5_subfamilies.html, updated to 03/June/2016) as references. The sequences were aligned using the MAFFT online service (Katoh and Toh, 2008) (http://mafft.cbrc.jp/alignment/server/). A neighbour‐joining tree was constructed using an integrated online tool provided by MAFFT as previously described (Tan *et al*., 2016a,b).

The phylogenetic trees of 16S rRNA genes and the putative GH5_46 proteins were respectively visualized and exported using the Interactive Tree Of Life (iTOL) as previously described (Tan *et al*., 2013).

### Overexpression of CbGH5 in *P. pastoris*

The coding region of *cbGH5*, excluding the predicted signal peptide, was cloned into a pPIC9K vector (Invitrogen) between the *Eco*RI and *Not*I sites. An α‐factor signal peptide provided by the pPIC9k vector was in‐frame fused to the N‐terminal of CbGH5 for secreting expression. An 8× histidine tag was in‐frame fused to the C‐terminal to facilitate the following purification.

The constructed recombinant plasmid pPIC9K‐CbGH5 was transformed into *P. pastoris* GS115 cells (Invitrogen). The transformed *P. pastoris* clone was overexpressed in five flasks. For each flask, the clone was initially grown in 5 ml of YPD medium at 28 °C with 250 rpm shaking for 24 h, and then inoculated into 1000 ml of BMGY medium (in a 5000 ml baffled flask). The culture was then grown at 28 °C with 250 rpm shaking for 48 h, then resuspended in 500 ml of BMMY medium (in a 5000 ml baffled flask) containing 1.0% methanol. The culture was further induced for 4 days, by supplementing methanol to a final concentration of 1.0% every 24 h. After 4 days, the culture with a total volume of 5 × 500 ml was harvested.

### Purification of CbGH5

CbGH5 was initially purified by Ni‐affinity chromatography and further refined twice by gel‐filtration (Table S1), similarly as the procedures described previously (Tan *et al*., 2016a,b, 2017). All purification steps were carried out at 4 °C. The 2500 ml of culture was centrifuged at 10 000 *g* for 30 min. The supernatant of about 2400 ml was collected, combined and concentrated to about 400 ml using a Pellicon ultrafiltration system (Merck Millipore, Billerica, MA, USA). The molecular weight cut‐off (MWCO) of the ultrafiltration membrane was 5 kDa. For the Ni‐affinity chromatography, the concentrated supernatant was loaded onto a HisTrap Excel column (GE Healthcare, Pittsburgh, PA, USA) prepacked with 5 ml of Ni‐sepharose excel resin. The column was pre‐equilibrated by buffer PE (20 mM KH_2_PO_4_‐K_2_HPO_4_ buffer (PBS), 100 mM NaCl, at pH 7.4). The column was then washed with 100 ml of buffer W1 (20 mM PBS, 100 mM NaCl, 5 mM imidazole, at pH 7.4) and 10 ml of buffer W2 (20 mM PBS, 100 mM NaCl, 150 mM imidazole, at pH 7.4). The elution step used buffer EL (20 mM PBS, 100 mM NaCl, 300 mM imidazole, at pH 7.4). The target protein CbGH5 was eluted and collected in about 28 ml. The 28 ml was concentrated to 0.3 ml with ultrafiltration spin‐columns with a 3 kDa MWCO (Merck‐Millipore). The protein sample was then de‐glycosylated with endoglycosidase H (Endo H) (New England Biolabs, Ipswich, MA, USA) using the method previously described by Yao *et al*. (2013) and Sakai *et al*. (2017). After de‐glycosylation, the protein sample was refined twice by gel‐filtration. The sample was loaded onto a Superdex 200 10/300GL gel‐filtration column (GE Healthcare) which had been pre‐equilibrated by buffer GF (10 mM Tris–HCl at pH 7.4). Buffer GF was also used for elution, at a flow rate of 0.5 ml min^−1^. About 1.5 ml of elution product was obtained from the first gel‐filtration. The 1.5 ml was then divided as 3 × 0.5 ml fractions. Each 0.5 ml was loaded again onto the Superdex 200 10/300GL gel‐filtration column and eluted at a flow rate of 0.5 ml min^−1^. About 3.2 ml of elution product was obtained.

The Mw and homogeneity of the purified protein were determined with MALDI‐TOF analysis by Sangon Biotech Inc. using an AB Sciex 5800 MALDI‐TOF mass spectrometer system (Applied Biosystems, Foster, CA, USA), as previously described (Tan *et al*., 2016a,b). The purified CbGH5 was quantified by Bradford colorimetric assay (Bradford, 1976) using bovine serum albumin as a calibrating standard as previously described (Tan *et al*., 2016a,b). The protein was then adjusted to a final concentration of 2 mg ml^−1^, frozen by liquid nitrogen and kept in a −80 °C freezer until used.

### Measuring enzyme activity on different substrates

Hydrolytic activities on several different substrates were initially assayed at pH 7, 60 °C. The suppliers of the chemicals were listed in Table S2. All the assays were made in triplicate, using 50 ng of CbGH5 in a 1 ml enzymatic reaction. For each assay, the procedures described by Jagtap *et al*. (2014) were used to measure the release of glucose, xylose or mannose. One unit (U) of activity is defined as the amount of enzyme needed for releasing 1 μmol of reducing monosaccharide (glucose, xylose or mannose, in this study) per minute.

### Determination of pH and temperature profiles

EG‐CMC and XYN‐bw activities were chosen to represent the cellulase and xylanase activities of CbGH5. For pH profile, the EG‐CMC and XYN‐bw activities were measured at different pH at 90 °C. The follow buffers were used at a final concentration of 200 mM: citric acid buffer for pH 3–6, Tris–HCl buffer for pH 7–9 and NaHCO_3_‐Na_2_CO_3_ buffer for pH 10–12. For temperature profile, EG‐CMC and XYN‐bw activities were measured at 60, 70, 80, 90 and 100 °C at their respective optimum pH.

### Determination of pH and temperature stability

To determine the pH stability, CbGH5 was incubated in buffers at pH 4, 8, 9 or 11 for 0–300 min at 90 °C, and then the residual EG‐CMC and XYN‐bw activities were measured under their respective optimum conditions. Decline curves of the activities were drawn. *t*_1/2_ was deduced by extrapolating from the 50% residual activity point on the decline curve. To determine the temperature stability, the enzyme was incubated in waterbath of 60, 70, 80, 90 or 100 °C for 0–300 min, at pH 9 for the EG‐CMC activity and 8 for XYN‐bw. Decline curves and *t*_1/2_ were obtained in the same way.

### Determination of enzyme kinetics

Kinetic parameters of the EG‐CMC and XYN‐bw activities of CbGH5 were determined respectively at their optimum pH and temperatures. Activity was measured (in triplicate) over a range of substrate concentrations (0.1–10.0 mg ml^−1^). Kinetic constants (*K*_m_ and *V*_max_) were estimated by Eadie‐Hofstee plot (Fig. S7) as previously described (Tan *et al*., 2016a,b). Catalytic turnover number (*k*_cat_) was calculated using an equation *k*_cat_ = *V*_max_/[E] (Guo *et al*., 2009), in which [E] means the molar concentration of the enzyme calculated based on its mass concentration and Mw. Catalytic efficiency (*k*_cat_ *K*_m_^−1^) was then calculated.

### Assessing the effects of cations, chelating agents and denaturants

To assess the cation effects, each cation was supplemented into the enzyme activity assay, and the residual EG‐CMC and XYN‐bw activities were measured (in triplicate) at respective optimum pH and temperature. The measured activity under the influence of each cation is shown in percentage, while the activity without adding any cation is defined as 100%.

To assess the effects of chelating agents and denaturants, the residual activities of EG‐CMC and XYN‐bw were measured when EDTA and EGTA were supplemented at 5 and 20 mM concentrations, SDS supplemented at 0.1%, 1% and 10%, urea supplemented at 5, 100 and 5 M respectively.

### Structural modelling

The structural model of the recombinant CbGH5 was predicted by NovaFold (version 14) of LaserGene 14 (DNASTAR Inc.) using the I‐TASSER (Iterative Threading ASSEmbly Refinement) algorithm (Yang *et al*., 2015), which constructs the model by threading through multiple homologue templates plus *ab initio* folding simulation of unmapped section. The quality of modelling was examined using the methods described by Shivange *et al*. (2012). The model was visualized with UCSF Chimera version 1.10.2 (Pettersen *et al*., 2004).

### Construction of CbGH5ΔCBM6 mutant

The CbGH5ΔCBM6 mutant was constructed by truncating CbGH5 between the Gln419 and Val420 residues and thus deleting the CBM6 domain. The fragment excluding the CBM6 domain was amplified by PCR and ligated into the pPIC9K vector. The CbGH5ΔCBM6 protein was overexpressed in *P. pastoris* GS115 and purified with the same procedures as CbGH5.

### Site‐directed mutagenesis

For protein with mutations in less than three sites, the plasmid was constructed by PCR‐based mutagenesis using a MutanBEST Kit (TaKaRa, Dalian, China) following the instruction provided by the manufacturer. For protein with multiple‐site (≥ 3 AA) mutations, the plasmid was constructed by directly chemical synthesis (by Genewiz Biotechnology Inc., Suzhou, China).

The mutant plasmids were verified by DNA sequencing and transformed into *P. pastoris* GS115. The mutant proteins were overexpressed, purified and characterized using the same procedures mentioned above.

### Preparation of straw and spent mushroom substrate

Wheat straw, rice straw, corn stalk and oilseed rape stalk were collected from Jianyang area, Sichuan Province, China. The spent mushroom substrate used in this study was collected from a mushroom farmer in Jianyang, Sichuan Province, China. The recipe of the mushroom cultivation substrate was: 80% (w w^−1^) straw, 10% (w w^−1^) cotton seed hulls, 8% (w w^−1^) wheat bran and 2% (w w^−1^) quicklime (calcium oxide). The straws were cut to a length of 2–3 cm, mixed with other ingredients and sterilized by steaming for 20 h at 100 °C before inoculation of oyster mushroom. The variety of the *Pleurotus ostreastus* used for this study was Gao‐Ping, a common commercial variety of oyster mushroom in China. Spent mushroom substrates made from the four crop straws were collected respectively after the oyster mushroom was harvested.

After collection, each of the materials was sun‐dried to constant weight, cut and homogenized to powder by a food processor and passed through a 40 mesh sieve.

### Saccharification of straw and spent mushroom substrate

For saccharification, 1 g of the straw powder or the spent mushroom substrate powder was soaked in 20 ml of Tris–HCl buffer (pH 8.5), added with 0.11 mg of CbGH5 [equal to 356 U of EG‐CMC, 15 U of filter paper cellulase (FPU) and 200 U of xylanase] and incubated at 90 °C, pH 8.5 for 2 h with gent shaking. The saccharification assay was performed in triplicate.

For comparison, commercial cellulase (catalog code NS50013, 70 FPU g^−1^, Novozymes A/S; Beijing, China) plus xylanase (Novozymes A/S, Beijing, China, NS50014, 600 U g^−1^) was used to replace CbGH5 in the assay. Twenty FPU of the Novozymes NS50013 cellulase plus 200 U of the NS50014 xylanase was used in 1 g of straw or spent mushroom substrate, as previously adopted by Zhu *et al*. (2013). The condition of the enzymatic reaction was pH 4.8, 45 °C, near the optimum pH and temperature for the mixed NS50013 + NS50014 enzymes as previously used (Zhu *et al*., 2013; Kafle *et al*., 2015).

### Composition analysis

Straw and spent mushroom substrate before (raw material) or after saccharification (hydrolysate) were subjected to composition analysis. Contents of lignin, cellulose, hemicellulose, free glucose and free xylose were measured using the procedures described in the National Renewable Energy Laboratory (NREL) standard method (Sluiter *et al*., 2008). In this method, lignin was acetylated and then quantified based on the optical absorbance at 280 nm, while cellulose, hemicellulose, free glucose and free xylose were quantified with an HPLC system (Shimadzu, Japan) installed with an Aminex HPX‐87P column (Bio‐Rad, USA). About 0.00004% (w/v) sulphuric acid was used as a mobile phase with a flow rate at 0.6 ml min^−1^, and the column temperature was 65 °C, as previously adopted by Zhu *et al*. (2013).

### Statistical analysis

Significance of difference was assessed by one‐way ANOVA, using PASW Statistics version 18 (IBM Corporation, Armonk, NY, USA).

### Availability and accession of strain and nucleotide sequences

*Chryseobacterium* sp. HT1 has been deposited to the China General Microbiological Culture Collection Center (CGMCC) under the accession number CGMCC1.15712. The 16S rRNA gene fragment has been deposited to GenBank under the accession number KX101126. The 4643 bp fragment obtained by genome walking has been deposited to GenBank under the accession number KX101127. The protein sequence of CbGH5 is accessible with GenBank accession number ANQ80467.

## Conflict of interest

None declared.

## Ethical statement

This article does not contain any studies with human participants or animals performed by any of the authors. This is the original work of the authors. The work described has not been submitted elsewhere for publication, in whole or in part. All authors confirm that ethical principles have been followed in the research as well as in manuscript preparation, and approved this submission.

## Supporting information


**Fig. S1.** Colonies of *Chryseobacterium* sp. HT1 on a LB agar plate.
**Fig. S2.** Alignment of the AA sequences of CbGH5 with 70 putative proteins of GH5_46 (partially shown). The two conserved glutamate residues which were believed to be the catalytic center were pointed by red triangles.
**Fig. S3.** (A) SDS‐PAGE analysis of CbGH5. Lane 1: Precision Plus Protein Dual Color Marker (Bio‐Rad). Lane 2: Purified CbGH5. **(B)** MALDI‐TOF analysis showed that the Mw of CbGH5 was 65892.97 Da and the protein was purified to homogeneity.
**Fig. S4.** Templates used by NovaFold for threading the structural model of CbGH5. The mapped and threaded sections were highlighted in red.
**Fig. S5.** Comparison of the structural models of the CbGH5 wild‐type (A) and the CbGH5(I107G, L109G, L153G, L155G, L157G, F257G, F280G, F282G) multiple‐site mutant (B), showing that the overall skeletons are similar. Both structural models were predicted by NovaFold as described in the Experimental procedures section.
**Fig. S6.** Degenerate primers CL‐F and CL‐R for amplifying the core region of putative cellulase gene. Designed based on conserved sequences presented in the alignment of 27 putative cellulase genes of *Chryseobacterium* microbes.
**Fig. S7.** Eadie‐Hofstee plot (three replicates) to determine the *K*_*m*_ and *V*_*max*_ of the EG‐CMC (A) and XYN‐bw (B) activities of CbGH5.
**Table S1.** Purification summary.
**Table S2.** Summary of substrate tested and the chemicals used.
**Table S3.** Primers for cloning *cbGH5* and flanking regions.
**Table S4.** Impact of site‐mutagenesis at Glu215 and Glu312 on the EG‐CMC and XYN‐bw activities.
**Table S5.** Activity levels and kinetic parameters of CbGH5 and CbGH5ΔCBM6.
**Table S6.** Lignin, cellulose, hemicellulose, glucose and xylose contents in straws and spent mushroom substrates.

## References

[mbt213034-bib-0001] Ariza, A. , Moroz, O.V. , Blagova, E.V. , Turkenburg, J.P. , Waterman, J. , Roberts, S.M. , *et al* (2013) Degradation of phytate by the 6‐phytase from *Hafnia alvei*: a combined structural and solution study. PLoS ONE 8: e65062. 23741456 10.1371/journal.pone.0065062 PMC3669009

[mbt213034-bib-0002] Assareh, R. , Shahbani Zahiri, H. , Akbari Noghabi, K. , Aminzadeh, S. and Bakhshi khaniki, G. (2012) Characterization of the newly isolated *Geobacillus* sp. T1, the efficient cellulase‐producer on untreated barley and wheat straws. Bioresour Technol 120, 99–105. 22784959 10.1016/j.biortech.2012.06.027

[mbt213034-bib-0003] Bischoff, K.M. , Rooney, A.P. , Li, X.‐L. , Liu, S. , and Hughes, S.R. (2006) Purification and characterization of a family 5 endoglucanase from a moderately thermophilic strain of *Bacillus licheniformis* . Biotechnol Lett 28: 1761–1765. 16900329 10.1007/s10529-006-9153-0

[mbt213034-bib-0004] Borges, T.A. , Souza, A.T.d. , Squina, F.M. , Riaño‐Pachón, D.M. , Santos, R.A.C.d. , Machado, E. , *et al* (2014) Biochemical characterization of an endoxylanase from *Pseudozyma brasiliensis* sp. nov. strain GHG001 isolated from the intestinal tract of Chrysomelidae larvae associated to sugarcane roots. Process Biochem 49, 77–83.

[mbt213034-bib-0005] Bradford, M.M. (1976) A rapid and sensitive method for the quantization of microgram quantities of protein utilizing the principle of protein‐dye binding. Anal Biochem 72: 248–254. 942051 10.1016/0003-2697(76)90527-3

[mbt213034-bib-0006] Brigidi, P. , Swennen, E. , Vitali, B. , Rossi, M. , and Matteuzzi, D. (2003) PCR detection of *Bifidobacterium* strains and *Streptococcus thermophilus* in feces of human subjects after oral bacteriotherapy and yogurt consumption. Int J Food Microbiol 81: 203–209. 12485746 10.1016/s0168-1605(02)00245-3

[mbt213034-bib-0007] Chang, L. , Ding, M. , Bao, L. , Chen, Y. , Zhou, J. , and Lu, H. (2011) Characterization of a bifunctional xylanase/endoglucanase from yak rumen microorganisms. Appl Microbiol Biotechnol 90: 1933. 21455595 10.1007/s00253-011-3182-x

[mbt213034-bib-0008] Cheng, J. , Huang, S. , Jiang, H. , Zhang, Y. , Li, L. , Wang, J. , and Fan, C. (2015) Isolation and characterization of a non‐specific endoglucanase from a metagenomic library of goat rumen. World J Microbiol Biotechnol 32: 12. 26712627 10.1007/s11274-015-1957-4

[mbt213034-bib-0009] Choi, J.‐H. , Lee, O.‐S. , Shin, J.‐H. , KWAK, Y.‐Y. , Kim, Y.‐M. and Rhee, I.‐K. (2006) Thermostable xylanase encoded by xynA of *Streptomyces thermocyaneoviolaceus*: cloning, purification, characterization and production of xylooligosaccharides. J Microbiol Biotechnol 16, 57–63.

[mbt213034-bib-0010] Claesson, M.J. , Jeffery, I.B. , Conde, S. , Power, S.E. , O'Connor, E.M. , Cusack, S. , *et al* (2012) Gut microbiota composition correlates with diet and health in the elderly. Nature 488: 178–184. 22797518 10.1038/nature11319

[mbt213034-bib-0011] Correia, M.A.S. , Mazumder, K. , Brás, J.L.A. , Firbank, S.J. , Zhu, Y. , Lewis, R.J. , *et al* (2011) Structure and function of an arabinoxylan‐specific xylanase. J Biol Chem 286: 22510–22520. 21378160 10.1074/jbc.M110.217315 PMC3121396

[mbt213034-bib-0012] Cutfield, S.M. , Davies, G.J. , Murshudov, G. , Anderson, B.F. , Moody, P.C.E. , Sullivan, P.A. , and Cutfield, J.F. (1999) The structure of the exo‐β‐(1,3)‐glucanase from *Candida albicans* in native and bound forms: relationship between a pocket and groove in family 5 glycosyl hydrolases1. J Mol Biol 294: 771–783. 10610795 10.1006/jmbi.1999.3287

[mbt213034-bib-0013] DeCastro, M.‐E. , Rodríguez‐Belmonte, E. and González‐Siso, M.‐I. (2016) Metagenomics of thermophiles with a focus on discovery of novel thermozymes. Front 7, 1521. 10.3389/fmicb.2016.01521 PMC5037290 27729905

[mbt213034-bib-0014] Ding, M. , Teng, Y. , Yin, Q. , Zhao, J. , and Zhao, F. (2008) The N‐terminal cellulose‐binding domain of EGXA increases thermal stability of xylanase and changes its specific activities on different substrates. Acta Biochim Biophys Sin 40: 949–954. 18989576 10.1111/j.1745-7270.2008.00481.x

[mbt213034-bib-0015] Dominguez, R. , Souchon, H. , Spinelli, S. , Dauter, Z. , Wilson, K.S. , Chauvaux, S. , *et al* (1995) A common protein fold and similar active site in two distinct families of beta‐glycanases. Nat Struct Biol 2: 569–576. 7664125 10.1038/nsb0795-569

[mbt213034-bib-0016] Ducros, V. , Czjzek, M. , Belaich, A. , Gaudin, C. , Fierobe, H.‐P. , Belaich, J.‐P. , *et al* (1995) Crystal structure of the catalytic domain of a bacterial cellulase belonging to family 5. Structure 3: 939–949. 8535787 10.1016/S0969-2126(01)00228-3

[mbt213034-bib-0017] Elisashvili, V. , Kachlishvili, E. , and Penninckx, M. (2008) Lignocellulolytic enzymes profile during growth and fruiting of *Pleurotus ostreatus* on wheat straw and tree leaves. Acta Microbiol Immunol Hung 55: 157–168. 18595320 10.1556/AMicr.55.2008.2.7

[mbt213034-bib-0018] Ghatge, S.S. , Telke, A.A. , Kang, S.‐H. , Arulalapperumal, V. , Lee, K.‐W. , Govindwar, S.P. , *et al* (2014) Characterization of modular bifunctional processive endoglucanase Cel5 from *Hahella chejuensis* KCTC 2396. Appl Microbiol Biotechnol 98: 4421–4435. 24343767 10.1007/s00253-013-5446-0

[mbt213034-bib-0019] Guo, B. , Chen, X.‐L. , Sun, C.‐Y. , Zhou, B.‐C. , and Zhang, Y.‐Z. (2009) Gene cloning, expression and characterization of a new cold‐active and salt‐tolerant endo‐β‐1,4‐xylanase from marine *Glaciecola mesophila* KMM 241. Appl Microbiol Biotechnol 84: 1107–1115. 19506861 10.1007/s00253-009-2056-y

[mbt213034-bib-0020] Gupta, A. , and Verma, J.P. (2015) Sustainable bio‐ethanol production from agro‐residues: a review. Renew Sust Energ Rev 41: 550–567.

[mbt213034-bib-0021] Henshaw, J.L. , Bolam, D.N. , Pires, V.M.R. , Czjzek, M. , Henrissat, B. , Ferreira, L.M.A. , *et al* (2004) The family 6 carbohydrate binding module cmCBM6‐2 contains two ligand‐binding sites with distinct specificities. J Biol Chem 279: 21552–21559. 15004011 10.1074/jbc.M401620200

[mbt213034-bib-0022] Hess, M. , Sczyrba, A. , Egan, R. , Kim, T.W. , Chokhawala, H. , Schroth, G. , *et al* (2011) Metagenomic discovery of biomass‐degrading genes and genomes from cow rumen. Science 331: 463–467. 21273488 10.1126/science.1200387

[mbt213034-bib-0023] Höcker, B. , Jürgens, C. , Wilmanns, M. , and Sterner, R. (2001) Stability, catalytic versatility and evolution of the (βα)8‐barrel fold. Curr Opin Biotechnol 12: 376–381. 11551466 10.1016/s0958-1669(00)00230-5

[mbt213034-bib-0024] Holmes, B. , Steigerwalt, A.G. , and Nicholson, A.C. (2013) DNA‐DNA hybridization study of strains of *Chryseobacterium*,* Elizabethkingia* and *Empedobacter* and of other usually indole‐producing non‐fermenters of CDC groups IIc, IIe, IIh and IIi, mostly from human clinical sources, and proposals of *Chryseobacterium bernardetii* sp. nov., *Chryseobacterium carnis* sp. nov., *Chryseobacterium lactis* sp. nov., *Chryseobacterium nakagawai* sp. nov. and *Chryseobacterium taklimakanense* comb. nov. Int J Syst Evol Microbiol 63: 4639–4662. 23934253 10.1099/ijs.0.054353-0 PMC4626006

[mbt213034-bib-0025] Jagtap, S.S. , Dhiman, S.S. , Kim, T.‐S. , Kim, I.‐W. , and Lee, J.‐K. (2014) Characterization of a novel endo‐β‐1,4‐glucanase from *Armillaria gemina* and its application in biomass hydrolysis. Appl Microbiol Biotechnol 98: 661–669. 23604561 10.1007/s00253-013-4894-x

[mbt213034-bib-0026] Jang, H.‐D. , and Chen, K.‐S. (2003) Production and characterization of thermostable cellulases from *Streptomyces* transformant T3‐1. World J Microbiol Biotechnol 19: 263–268.

[mbt213034-bib-0027] Jia, X. , Qiao, W. , Tian, W. , Peng, X. , Mi, S. , Su, H. , and Han, Y. (2016) Biochemical characterization of extra‐ and intracellular endoxylanse from thermophilic bacterium *Caldicellulosiruptor kronotskyensis* . Sci Rep 6: 21672. 26899227 10.1038/srep21672 PMC4761950

[mbt213034-bib-0028] Jiang, Y. , Duarte, A.V. , van den Brink, J. , Wiebenga, A. , Zou, G. , Wang, C. , *et al* (2016) Enhancing saccharification of wheat straw by mixing enzymes from genetically‐modified *Trichoderma reesei* and *Aspergillus niger* . Biotechnol Lett 38: 65–70. 26354856 10.1007/s10529-015-1951-9 PMC4706842

[mbt213034-bib-0029] Kafle, K. , Shin, H. , Lee, C.M. , Park, S. , and Kim, S.H. (2015) Progressive structural changes of Avicel, bleached softwood, and bacterial cellulose during enzymatic hydrolysis. Sci Rep 5: 15102. 26463274 10.1038/srep15102 PMC4604514

[mbt213034-bib-0030] Kämpfer, P. , Dreyer, U. , Neef, A. , Dott, W. , and Busse, H.‐J. (2003) *Chryseobacterium defluvii* sp. nov., isolated from wastewater. Int J Syst Evol Microbiol 53: 93–97. 12656158 10.1099/ijs.0.02073-0

[mbt213034-bib-0031] Kämpfer, P. , Poppel, M.T. , Wilharm, G. , Busse, H.‐J. , McInroy, J.A. , and Glaeser, S.P. (2014) *Chryseobacterium gallinarum* sp. nov., isolated from a chicken, and *Chryseobacterium contaminans* sp. nov., isolated as a contaminant from a rhizosphere sample. Int J Syst Evol Microbiol 64: 1419–1427. 24449786 10.1099/ijs.0.058933-0

[mbt213034-bib-0032] Karnaouri, A.C. , Topakas, E. , and Christakopoulos, P. (2014) Cloning, expression, and characterization of a thermostable GH7 endoglucanase from *Myceliophthora thermophila* capable of high‐consistency enzymatic liquefaction. Appl Microbiol Biotechnol 98: 231–242. 23615741 10.1007/s00253-013-4895-9

[mbt213034-bib-0033] Katoh, K. , and Toh, H. (2008) Recent developments in the MAFFT multiple sequence alignment program. Brief Bioinform 9: 286–298. 18372315 10.1093/bib/bbn013

[mbt213034-bib-0034] Kim, J.S. , Lee, Y.Y. , and Kim, T.H. (2016) A review on alkaline pretreatment technology for bioconversion of lignocellulosic biomass. Bioresour Technol 199: 42–48. 26341010 10.1016/j.biortech.2015.08.085

[mbt213034-bib-0035] Kitago, Y. , Karita, S. , Watanabe, N. , Kamiya, M. , Aizawa, T. , Sakka, K. , and Tanaka, I. (2007) Crystal structure of Cel44A, a glycoside hydrolase family 44 endoglucanase from *Clostridium thermocellum* . J Biol Chem 282: 35703–35711. 17905739 10.1074/jbc.M706835200

[mbt213034-bib-0036] Lafond, M. , Guais, O. , Maestracci, M. , Bonnin, E. , and Giardina, T. (2014) Four GH11 xylanases from the xylanolytic fungus *Talaromyces versatilis* act differently on (arabino)xylans. Appl Microbiol Biotechnol 98: 6339–6352. 24664446 10.1007/s00253-014-5606-x

[mbt213034-bib-0037] Lane, D.J. (1991) 16S/23S rRNA sequencing In Nucleic Acid Techniques in Bacterial Systematics. Stackebrandt E., and Goodfellow M. (eds). Chichester, UK: John Wiley & Sons, pp. 115–147.

[mbt213034-bib-0038] Le, Y. , and Wang, H. (2014) High‐level soluble expression of a thermostable xylanase from thermophilic fungus *Thermomyces lanuginosus* in *Escherichia coli* via fusion with OsmY protein. Protein Expres Purif 99: 1–5. 10.1016/j.pep.2014.03.004 24657705

[mbt213034-bib-0039] Li, P.‐p. , Wang, X.‐j. , Yuan, X.‐f. , Wang, X.‐f. , Cao, Y.‐z. and Cui, Z.‐j. (2011) Screening of a composite microbial system and its characteristics of wheat straw degradation. Agric Sci China 10, 1586–1594.

[mbt213034-bib-0040] Li, J. , Zhang, H. , Wu, M. , Wang, C. , Dong, Y. , Zhu, L. , and Zhang, P. (2014) Expression and characterization of hyperthermotolerant Xylanases, SyXyn11P and SyXyn11E, in *Pichia pastoris* and *Escherichia coli* . Appl Biochem Biotechnol 172: 3476–3487. 24549804 10.1007/s12010-014-0786-5

[mbt213034-bib-0041] Libo, Z. , Yongqiang, L. and Lu, H. (2016) Contributions of open crop straw burning emissions to PM 2.5 concentrations in China. Environ Res Lett 11, 014014.

[mbt213034-bib-0042] Liu, Y. , Dun, B. , Shi, P. , Ma, R. , Luo, H. , Bai, Y. , *et al* (2015) A novel GH7 endo‐β‐1,4‐glucanase from *Neosartorya fischeri* P1 with good thermostability, broad substrate specificity and potential application in the brewing industry. PLoS ONE 10: e0137485. 26360701 10.1371/journal.pone.0137485 PMC4567307

[mbt213034-bib-0043] Meng, X. , and Ragauskas, A.J. (2014) Recent advances in understanding the role of cellulose accessibility in enzymatic hydrolysis of lignocellulosic substrates. Curr Opin Biotechnol 27: 150–158. 24549148 10.1016/j.copbio.2014.01.014

[mbt213034-bib-0044] Nakazawa, H. , Okada, K. , Kobayashi, R. , Kubota, T. , Onodera, T. , Ochiai, N. , *et al* (2008) Characterization of the catalytic domains of *Trichoderma reesei* endoglucanase I, II, and III, expressed in *Escherichia coli* . Appl Microbiol Biotechnol 81: 681–689. 18762935 10.1007/s00253-008-1667-z

[mbt213034-bib-0045] Nam, K.H. , Lee, W.H. , Rhee, K.H. , and Hwang, K.Y. (2010) Structural characterization of the bifunctional glucanase–xylanase CelM2 reveals the metal effect and substrate‐binding moiety. Biochem Biophys Res Commun 391: 1726–1730. 20043877 10.1016/j.bbrc.2009.12.141

[mbt213034-bib-0046] O'Donnell, M.M. , Harris, H.M.B. , Jeffery, I.B. , Claesson, M.J. , Younge, B. , O’ Toole, P.W. and Ross, R.P. (2013) The core faecal bacterial microbiome of Irish Thoroughbred racehorses. Lett Appl Microbiol 57, 492–501. 23889584 10.1111/lam.12137

[mbt213034-bib-0047] Pellegrini, V.O.A. , Serpa, V.I. , Godoy, A.S. , Camilo, C.M. , Bernardes, A. , Rezende, C.A. , *et al* (2015) Recombinant *Trichoderma harzianum* endoglucanase I (Cel7B) is a highly acidic and promiscuous carbohydrate‐active enzyme. Appl Microbiol Biotechnol 99: 9591–9604. 26156238 10.1007/s00253-015-6772-1

[mbt213034-bib-0048] Petersen, T.N. , Brunak, S. , von Heijne, G. , and Nielsen, H. (2011) SignalP 4.0: discriminating signal peptides from transmembrane regions. Nat Methods 8: 785–786. 21959131 10.1038/nmeth.1701

[mbt213034-bib-0948] Pettersen, E.F. , Goddard, T.D. , Huang, C.C. , Couch, G.S. , Greenblatt, D.M. , Meng, E.C. , and Ferrin, T.E. (2004). UCSF Chimera–a visualization system for exploratory research and analysis. J Comput Chem 25: 1605–1612. 15264254 10.1002/jcc.20084

[mbt213034-bib-0049] Pollegioni, L. , Tonin, F. , and Rosini, E. (2015) Lignin‐degrading enzymes. FEBS J 282: 1190–1213. 25649492 10.1111/febs.13224

[mbt213034-bib-0050] Qiao, W. , Tang, S. , Mi, S. , Jia, X. , Peng, X. , and Han, Y. (2014) Biochemical characterization of a novel thermostable GH11 xylanase with CBM6 domain from *Caldicellulosiruptor kronotskyensis* . J Mol Cat B: Enz 107: 8–16.

[mbt213034-bib-0051] Rashamuse, K.J. , Visser, D.F. , Hennessy, F. , Kemp, J. , Roux‐van der Merwe, M.P. , Badenhorst, J. , *et al* (2013) Characterisation of two bifunctional cellulase–xylanase enzymes isolated from a bovine rumen metagenome library. Curr Microbiol 66: 145–151. 23086538 10.1007/s00284-012-0251-z

[mbt213034-bib-0052] Rattu, G. , Joshi, S. , and Satyanarayana, T. (2016) Bifunctional recombinant cellulase–xylanase (rBhcell‐xyl) from the polyextremophilic bacterium *Bacillus halodurans* TSLV1 and its utility in valorization of renewable agro‐residues. Extremophiles 20: 831–842. 27558695 10.1007/s00792-016-0870-6

[mbt213034-bib-0053] Sakai, K. , Mochizuki, M. , Yamada, M. , Shinzawa, Y. , Minezawa, M. , Kimoto, S. , *et al* (2017) Biochemical characterization of thermostable β‐1,4‐mannanase belonging to the glycoside hydrolase family 134 from *Aspergillus oryzae* . Appl Microbiol Biotechnol 101, 3237–3245. 28105485 10.1007/s00253-017-8107-x

[mbt213034-bib-0054] Shimada, T. , Yamazaki, Y. , Tanaka, K. , and Ishihama, A. (2014) The whole set of constitutive promoters recognized by rna polymerase RpoD holoenzyme of *Escherichia coli* . PLoS ONE 9: e90447. 24603758 10.1371/journal.pone.0090447 PMC3946193

[mbt213034-bib-0055] Shirkavand, E. , Baroutian, S. , Gapes, D.J. , and Young, B.R. (2016) Combination of fungal and physicochemical processes for lignocellulosic biomass pretreatment – A review. Renew Sust Energ Rev 54: 217–234.

[mbt213034-bib-0056] Shivange, A.V. , Serwe, A. , Dennig, A. , Roccatano, D. , Haefner, S. , and Schwaneberg, U. (2012) Directed evolution of a highly active *Yersinia mollaretii* phytase. Appl Microbiol Biotechnol 95: 405–418. 22159661 10.1007/s00253-011-3756-7

[mbt213034-bib-0057] Sluiter, A. , Hames, B. , Ruiz, R. , Scarlata, C. , Sluiter, J. , Templeton, D. and Crocker, D. (2008) Determination of structural carbohydrates and lignin in biomass. Laboratory Analytical Procedure (LAP). NREL/TP‐510‐42618. National Renewable Energy Laboratory, Golden, Colorado 80401.

[mbt213034-bib-0058] Solovyev, V. and Salamov, A. (2011) Automatic annotation of microbial genomes and metagenomic sequences In: Metagenomics and its Applications in Agriculture, Biomedicine and Environmental Studies. Li R.W. (ed). New York, NY: Nova Science Publishers, pp. 61–78.

[mbt213034-bib-0059] Sun, R. (2010) Cereal Straw as a Resource for Sustainable Biomaterials and Biofuels: Chemistry, Extractives, Lignins, Hemicelluloses and Cellulose. Radarweg, Netherland: Elsevier.

[mbt213034-bib-0060] Tan, H. , Barret, M. , Mooij, M.J. , Rice, O. , Morrissey, J.P. , Dobson, A.D. , *et al* (2013) Long‐term phosphorus fertilisation increased the diversity of the total bacterial community and the *phoD* phosphorus mineraliser group in pasture soils. Biol Fertility Soils 49: 661–672.

[mbt213034-bib-0061] Tan, H. , Miao, R. , Liu, T. , Cao, X. , Wu, X. , Xie, L. , *et al* (2016a) Enhancing thermal resistance of a novel *Acidobacteria*‐derived phytase by engineering of disulfide bridges. J Microbiol Biotechnol 26: 1717–1722. 27363471 10.4014/jmb.1604.04051

[mbt213034-bib-0062] Tan, H. , Wu, X. , Xie, L. , Huang, Z. , Peng, W. , and Gan, B. (2016b) Identification and characterization of a mesophilic phytase highly resilient to high‐temperatures from a fungus‐garden associated metagenome. Appl Microbiol Biotechnol 100: 2225–2241. 26536874 10.1007/s00253-015-7097-9

[mbt213034-bib-0063] Tan, H. , Tang, J. , Li, X. , Liu, T. , Miao, R. , Huang, Z. , *et al* (2017) Biochemical characterization of a psychrophilic phytase from an artificially cultivable morel *Morchella importuna* . J Microbiol Biotechnol https://doi.org/10.4014/jmb.1708.08007 (online, in press). 10.4014/jmb.1708.08007 29017237

[mbt213034-bib-0064] Taniguchi, M. , Suzuki, H. , Watanabe, D. , Sakai, K. , Hoshino, K. , and Tanaka, T. (2005) Evaluation of pretreatment with *Pleurotus ostreatus* for enzymatic hydrolysis of rice straw. J Biosci Bioeng 100: 637–643. 16473773 10.1263/jbb.100.637

[mbt213034-bib-0065] Wang, K. , Luo, H. , Bai, Y. , Shi, P. , Huang, H. , Xue, X. , and Yao, B. (2014) A thermophilic endo‐1,4‐β‐glucanase from *Talaromyces emersonii* CBS394.64 with broad substrate specificity and great application potentials. Appl Microbiol Biotechnol 98: 7051–7060. 24668246 10.1007/s00253-014-5680-0

[mbt213034-bib-0066] Wang, P. , Chang, J. , Yin, Q. , Wang, E. , Zhu, Q. , Song, A. , and Lu, F. (2015) Effects of thermo‐chemical pretreatment plus microbial fermentation and enzymatic hydrolysis on saccharification and lignocellulose degradation of corn straw. Bioresour Technol 194: 165–171. 26188559 10.1016/j.biortech.2015.07.012

[mbt213034-bib-0067] Wu, S. , Lan, Y. , Wu, Z. , Peng, Y. , Chen, S. , Huang, Z. , *et al* (2013) Pretreatment of spent mushroom substrate for enhancing the conversion of fermentable sugar. Bioresour Technol 148: 596–600. 24047726 10.1016/j.biortech.2013.08.122

[mbt213034-bib-0068] Yang, J. , Yan, R. , Roy, A. , Xu, D. , Poisson, J. , and Zhang, Y. (2015) The I‐TASSER Suite: protein structure and function prediction. Nat Methods 12: 7–8. 25549265 10.1038/nmeth.3213 PMC4428668

[mbt213034-bib-0069] Yao, M.Z. , Wang, X. , Wang, W. , Fu, Y.J. , and Liang, A.H. (2013) Improving the thermostability of *Escherichia coli* phytase, appA, by enhancement of glycosylation. Biotechnol Lett 35: 1669–1676. 23794051 10.1007/s10529-013-1255-x

[mbt213034-bib-0070] Yazawa, R. , Takakura, J. , Sakata, T. , Ihsanawati, Y. , R., F. and T., et al. (2011) A calcium‐dependent xylan‐binding domain of alkaline xylanase from alkaliphilic *Bacillus* sp. Strain 41M‐1. Biosci Biotechnol Biochem 75, 379–381. 21307573 10.1271/bbb.100730

[mbt213034-bib-0071] Yennamalli, R.M. , Rader, A.J. , Wolt, J.D. , and Sen, T.Z. (2011) Thermostability in endoglucanases is fold‐specific. BMC Struct Biol 11: 10. 21291533 10.1186/1472-6807-11-10 PMC3047435

[mbt213034-bib-0072] Yeoman, C.J. , Han, Y. , Dodd, D. , Schroeder, C.M. , Mackie, R.I. and Cann, I.K.O. (2010) Thermostable enzymes as biocatalysts in the biofuel industry. Adv Appl Microbiol Academic Press 70, 1–55. 20359453 10.1016/S0065-2164(10)70001-0 PMC4561533

[mbt213034-bib-0073] Young, C.‐C. , Kämpfer, P. , Shen, F.‐T. , Lai, W.‐A. , and Arun, A.B. (2005) *Chryseobacterium formosense* sp. nov., isolated from the rhizosphere of *Lactuca sativa* L. (garden lettuce). Int J Syst Evol Microbiol 55: 423–426. 15653912 10.1099/ijs.0.63331-0

[mbt213034-bib-0074] Yuan, S.‐F. , Wu, T.‐H. , Lee, H.‐L. , Hsieh, H.‐Y. , Lin, W.‐L. , Yang, B. , *et al* (2015) Biochemical characterization and structural analysis of a bifunctional cellulase/xylanase from *Clostridium thermocellum* . J Biol Chem 290: 5739–5748. 25575592 10.1074/jbc.M114.604454 PMC4342484

[mbt213034-bib-0075] Zhang, R. , Li, X. , and Fadel, J.G. (2002) Oyster mushroom cultivation with rice and wheat straw. Bioresour Technol 82: 277–284. 11991077 10.1016/s0960-8524(01)00188-2

[mbt213034-bib-0076] Zhang, C. , Zhang, W. , and Lu, X. (2015) Expression and characteristics of a Ca^2+^‐dependent endoglucanase from *Cytophaga hutchinsonii* . Appl Microbiol Biotechnol 99: 9617–9623. 26169628 10.1007/s00253-015-6746-3

[mbt213034-bib-0077] Zhu, H.‐J. , Liu, J.‐H. , Sun, L.‐F. , Hu, Z.‐F. , and Qiao, J.J. (2013) Combined alkali and acid pretreatment of spent mushroom substrate for reducing sugar and biofertilizer production. Bioresour Technol 136: 257–266. 23567689 10.1016/j.biortech.2013.02.121

