# Supplementary material for: A bifunctional cellulase–xylanase of a new Chryseobacterium strain isolated from the dung of a straw‐fed cattle
Source: Microb Biotechnol. 2017 Dec 4;11(2):381–98. doi: 10.1111/1751-7915.13034 (PMC5812240; doi:10.1111/1751-7915.13034)
Supplement: Supplementary file 1 — Fig. S1. Colonies of Chryseobacterium sp. HT1 on a LB agar plate. Fig. S2. Alignment of the AA sequences of CbGH5 with 70 putative proteins of GH5_46 (partially shown). The two conserved glutamate residues which were believed to be the catalytic center were pointed by red triangles. Fig. S3. (A) SDS‐PAGE analysis of CbGH5. Lane 1: Precision Plus Protein Dual Color Marker (Bio‐Rad). Lane 2: Purified CbGH5. (B) MALDI‐TOF analysis showed that the Mw of CbGH5 was 65892.97 Da and the protein was purified to homogeneity. Fig. S4. Templates used by NovaFold for threading the structural model of CbGH5. The mapped and threaded sections were highlighted in red. Fig. S5. Comparison of the structural models of the CbGH5 wild‐type (A) and the CbGH5(I107G, L109G, L153G, L155G, L157G, F257G, F280G, F282G) multiple‐site mutant (B), showing that the overall skeletons are similar. Both structural models were predicted by NovaFold as described in the Experimental procedures section. Fig. S6. Degenerate primers CL‐F and CL‐R for amplifying the core region of putative cellulase gene. Designed based on conserved sequences presented in the alignment of 27 putative cellulase genes of Chryseobacterium microbes. Fig. S7. Eadie‐Hofstee plot (three replicates) to determine the K m and V max of the EG‐CMC (A) and XYN‐bw (B) activities of CbGH5. Table S1. Purification summary. Table S2. Summary of substrate tested and the chemicals used. Table S3. Primers for cloning cbGH5 and flanking regions. Table S4. Impact of site‐mutagenesis at Glu215 and Glu312 on the EG‐CMC and XYN‐bw activities. Table S5. Activity levels and kinetic parameters of CbGH5 and CbGH5ΔCBM6. Table S6. Lignin, cellulose, hemicellulose, glucose and xylose contents in straws and spent mushroom substrates. [file MBT2-11-381-s001.docx]

**Supporting Information**

1. **Supplementary tables**

**Table S1.** Purification summary.

| **Step** | **Volume (ml)** | **Total protein (mg)** | **Total EG-CMC activity (U)** | **Phytase activity (at pH 7, 60 °C)** | **Yield (%)** |
| --- | --- | --- | --- | --- | --- |
| Culture supernatant | 2400 | - | 74431.2 | 31.0 U ml^-1^ | 100 |
| After concentrating by ultra-filtration | 400 | - | 67905.0 | 169.8 U ml^-1^ | 91.2 |
| After Ni-affinity chromatography | 28 | 17.2 | 51324.7 | 2979.1 U mg^-1^ | 69.0 |
| After gel-filtration | 3.2 | 6.8 | 21152.4 | 3102.4 U mg^-1^ | 28.4 |

**Table S2.** Summary of substrate tested and the chemicals used.

| Substrate used in the assay | Related activity | Biochemical relevance |
| --- | --- | --- |
| CMC-Na (Sigma, USA) | endoglucanase (*endo*-β-1,4-glucanase, EC 3.2.1.4) for CMC | hydrolase activity on amorphous cellulose |
| microcrystalline cellulose (Sigma, USA) | endoglucanase for microcrystalline cellulose (EC 3.2.1.4) | hydrolase activity on crystalline cellulose |
| filter paper (Sangon Biotech Inc., Shanghai, China) | filter paper activity (EC 3.2.1.4) | hydrolase activity on insoluble cellulose |
| β-D-glucan from barley (Sigma, USA) | endoglucanase for β-D-glucan (EC 3.2.1.4) | hydrolase activity on β-D-glucan |
| *p*-nitrophenyl β-D-glucoside (*p*NPG) (Sangon Biotech Inc., Shanghai, China) | β-glucosidase (EC 3.2.1.21) | hydrolase activity on terminal, non-reducing β-D-glucosyl residues |
| Azo-xylan (from birchwood) (Megazyme, Ireland) | xylanase (*endo*-1,4-β-D-xylanase, EC 3.2.1.8) | hydrolase activity on water-soluble xylan |
| corncob insoluble xylan (HarveyBio, China) | xylanase (*endo*-1,4-β-D-xylanase, EC 3.2.1.8) | hydrolase activity on insoluble xylan |
| locust bean galactomannan (LBG) (Sigma, USA) | mannanase (*endo*-1,4-β-D-mannanase, EC 3.2.1.78) | hydrolase activity on mannan |

**Table S3.** Primers for cloning *cbGH5* and flanking regions.

| **Primer name** | **Sequence (5’→3’)** | **Purpose** |
| --- | --- | --- |
| CL-F | TWHTWTCCGCHBTWTTATTGTCTCAATTTGGGACATC | degenerate PCR to obtain core region of *cbGH5* |
| CL-R | CCRTTRTARTTRTTDCCCCADCCGTTNCCTTC |  |
|  |  |  |
| gwCL-FSP1 | GATGCGATGTTCAGACAAGTGACAGATG | genome walking towards downstream, round 1 |
| gwCL-FSP2 | GAAGAATGGGCTCTGCTTATCTGG | genome walking towards downstream, round 2 |
| gwCL-FSP3 | TATCAATCTCTGGGTAAGTGATCCGGC | genome walking towards downstream, round 3 |
| gwCL-RSP1 | ATGCATAGGAAGCCTGATGGAGTTGAATC | genome walking towards upstream, round 1 |
| gwCL-RSP2 | TGCATGCCATCTTCGCCAATCAGCTC | genome walking towards upstream, round 2 |
| gwCL-RSP3 | TCCTTGATTTTATACTGAGGGCCGGC | genome walking towards upstream, round 3 |

**Table S4.** Impact of site-mutagenesis at Glu215 and Glu312 on the EG-CMC and XYN-bw activities.

| **Mutant** | **Substituted residue** | **EG-CMC activity** | **XYN-bw activity** |
| --- | --- | --- | --- |
| CbGH5 wild type | none | + | + |
| CbGH5(E215A) | Glu215→Ala | - | - |
| CbGH5(E312A) | Glu312→Ala | - | - |
| CbGH5(E215A,E312A) | Glu215→Ala, Glu312→Ala | - | - |

**Table S5.** Activity levels and kinetic parameters of CbGH5 and CbGH5ΔCBM6.

|  | **Substrates** | | | | | | | |
| --- | --- | --- | --- | --- | --- | --- | --- | --- |
|  | CMC | | **birchwood xylan** | | **filter paper** | | **corncob insoluble xylan** | |
|  | CbGH5 | CbGH5ΔCBM6 | CbGH5 | CbGH5ΔCBM6 | CbGH5 | CbGH5ΔCBM6 | CbGH5 | CbGH5ΔCBM6 |
| activity level (U mg^-1^) | 3237.15±160.23 | 2699.72±54.09 | 1792.83±115.65 | 1851.35±103.78 | 195.56±9.02 | 112.47±8.85 | 122.68±8.35 | 78.04±4.71 |
| *V_max_* (mM s^-1^) | 7.34±0.11 ×10^-5^ | 6.32±0.04 ×10^-5^ | 5.69±0.12 ×10^-5^ | 5.55±0.05 ×10^-5^ | N/A | N/A | N/A | N/A |
| *K_m_* (mg ml^-1^) | 1.80±0.03 | 2.01±0.03 | 4.52±0.11 | 4.00±0.07 | N/A | N/A | N/A | N/A |
| *k_cat_ K_m_^-1^* (ml mg^-1^ s^-1^) | 5.37±0.01 ×10^4^ | 4.14±0.05 ×10^4^ | 1.66±0.01 ×10^4^ | 1.83±0.02 ×10^4^ | N/A | N/A | N/A | N/A |

N/A: determination of kinetic parameters by Eadie–Hofstee plot not applicable for insoluble substrates

**Table S6.** Lignin, cellulose, hemicellulose, glucose and xylose contents in straws and spent mushroom substrates.

|  | | Content (%, w/w) | | | | |
| --- | --- | --- | --- | --- | --- | --- |
|  |  | lignin | cellulose | hemicellulose | glucose | xylose |
| wheat straw | before hydrolysis | 10.49±0.73 | 47.50±0.55 | 10.10±0.64 | 0.71±4.10E-3 | 1.21E-3±9.3E-5 |
|  | hydrolyzed by Novozymes cellulase+xylanase | 10.58±0.53 | 43.96±0.56 | 7.84±0.36 | 3.16±0.04 | 1.22±0.03 |
|  | hydrolyzed by CbGH5 | 10.34±0.65 | 33.39±0.41 | 6.98±0.75 | 12.98±0.12 | 2.17±0.06 |
|  |  |  |  |  |  |  |
| rice straw | before hydrolysis | 12.87±0.94 | 48.52±0.97 | 11.04±0.42 | 0.57±3.18E-3 | 9.28E-4±6.86E-5 |
|  | hydrolyzed by Novozymes cellulase+xylanase | 12.70±0.69 | 43.84±0.86 | 8.35±0.49 | 4.13±0.03 | 1.68±0.03 |
|  | hydrolyzed by CbGH5 | 13.09±0.63 | 34.21±0.61 | 6.72±0.42 | 12.93±0.16 | 2.32±0.06 |
|  |  |  |  |  |  |  |
| corn stalk | before hydrolysis | 11.62±0.79 | 45.48±0.70 | 8.09±0.20 | 0.60±4.57E-3 | 1.72E-3±2.90E-4 |
|  | hydrolyzed by Novozymes cellulase+xylanase | 11.21±1.05 | 41.24±0.36 | 5.90±0.75 | 3.76±0.03 | 1.68±0.02 |
|  | hydrolyzed by CbGH5 | 11.54±0.48 | 30.63±0.56 | 5.02±0.78 | 13.57±0.09 | 2.02±0.03 |
|  |  |  |  |  |  |  |
| oilseed stalk | before hydrolysis | 9.60±0.56 | 41.71±0.32 | 8.05±0.13 | 0.55±2.57E-3 | 5.34E-4±6.51E-5 |
|  | hydrolyzed by Novozymes cellulase+xylanase | 9.67±0.22 | 37.25±0.61 | 5.74±0.46 | 3.95±0.05 | 1.81±0.01 |
|  | hydrolyzed by CbGH5 | 9.46±0.34 | 26.11±0.40 | 4.57±0.58 | 13.90±0.11 | 2.53±0.09 |
|  |  |  |  |  |  |  |
| spent mushroom substrate from wheat straw | before hydrolysis | 21.31±0.69 | 51.37±0.86 | 11.44±0.15 | 0.38±0.02 | 0.17±0.02 |
|  | hydrolyzed by Novozymes cellulase+xylanase | 21.07±0.57 | 47.80±0.57 | 9.15±0.07 | 2.93±0.04 | 1.48±0.06 |
|  | hydrolyzed by CbGH5 | 21.20±0.87 | 9.11±0.74 | 5.69±0.11 | 40.29±0.60 | 5.78±0.32 |
|  |  |  |  |  |  |  |
| spent mushroom substrate from rice straw | before hydrolysis | 27.63±1.23 | 48.90±1.15 | 15.02±0.17 | 0.40±0.02 | 0.24±0.01 |
|  | hydrolyzed by Novozymes cellulase+xylanase | 27.79±0.50 | 45.45±0.83 | 12.85±0.08 | 2.82±0.08 | 1.41±0.04 |
|  | hydrolyzed by CbGH5 | 27.92±0.26 | 13.30±0.46 | 9.31±0.12 | 33.24±0.58 | 5.02±0.06 |
|  |  |  |  |  |  |  |
| spent mushroom substrate from corn stalk | before hydrolysis | 19.19±0.29 | 40.37±0.26 | 8.25±0.09 | 0.36±0.01 | 0.29±1.03E-3 |
|  | hydrolyzed by Novozymes cellulase+xylanase | 19.20±0.87 | 36.09±0.41 | 6.10±0.08 | 3.71±0.04 | 1.48±0.03 |
|  | hydrolyzed by CbGH5 | 19.00±0.38 | 5.89±0.77 | 2.09±0.12 | 31.13±0.83 | 5.36±0.08 |
|  |  |  |  |  |  |  |
| spent mushroom substrate from oilseed stalk | before hydrolysis | 24.44±0.56 | 48.44±0.34 | 12.80±0.11 | 0.37±2.52E-3 | 0.23±0.02 |
|  | hydrolyzed by Novozymes cellulase+xylanase | 24.61±0.58 | 44.95±0.70 | 10.53±0.07 | 2.96±0.06 | 1.51±0.05 |
|  | hydrolyzed by CbGH5 | 24.54±0.68 | 15.36±0.23 | 6.35±0.11 | 30.21±0.58 | 4.68±0.10 |

**3. Supplementary figures**


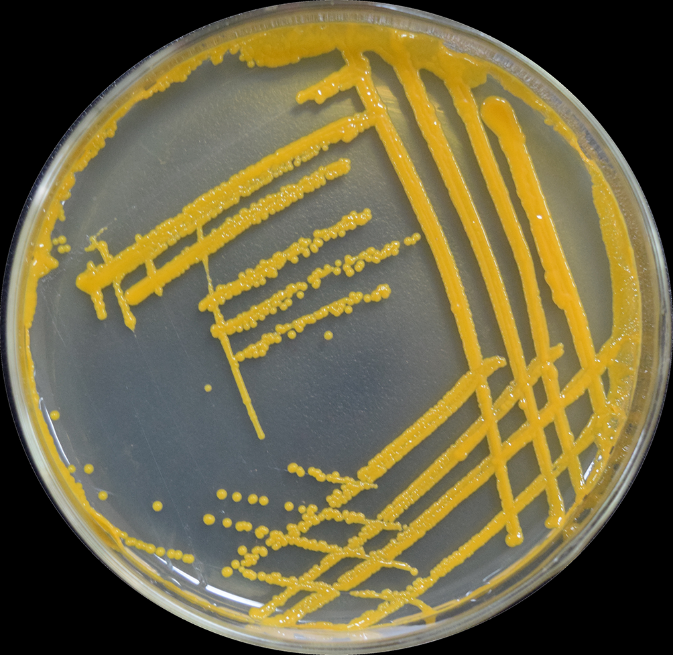


**Fig. S1. Colonies of *Chryseobacterium* sp. HT1 on a LB agar plate.**


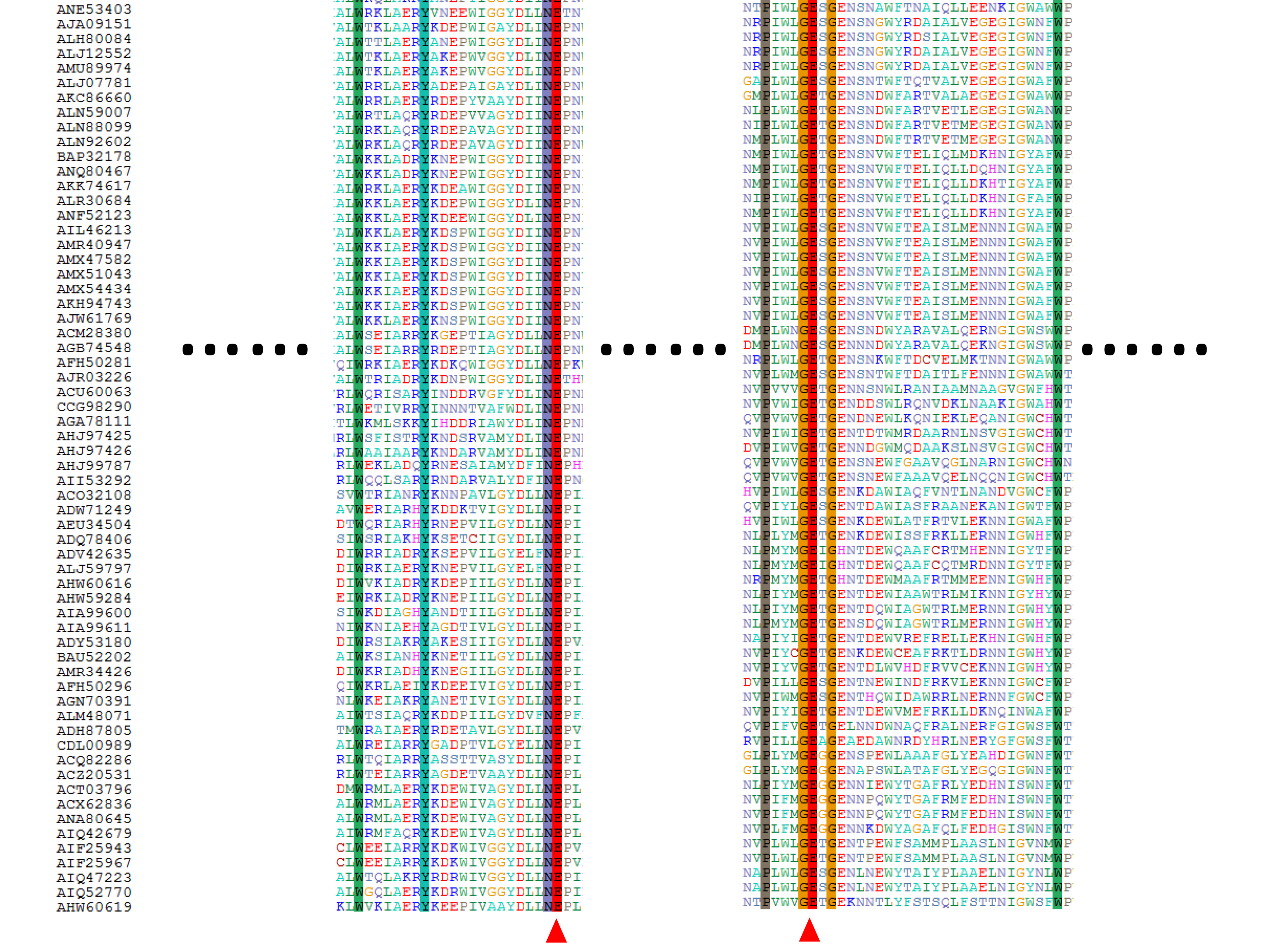


**Fig. S2.** Alignment of the AA sequences of CbGH5 with 70 putative proteins of GH5_46 (partially shown). The two conserved glutamate residues which were believed to be the catalytic center were pointed by red triangles.

**
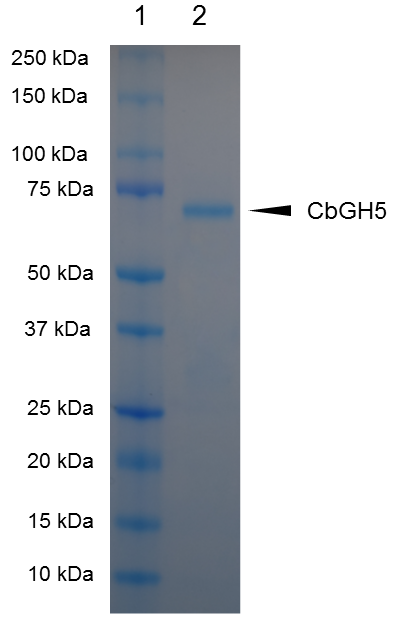
A B**


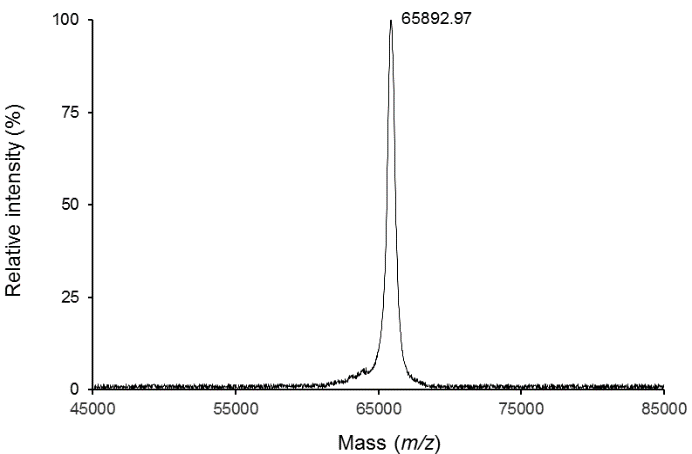


**Fig. S3.** **(A)** SDS-PAGE analysis of CbGH5. Lane 1: Precision Plus Protein Dual Color Marker (Bio-Rad, USA). Lane 2: Purified CbGH5. **(B)** MALDI-TOF analysis showed that the Mw of CbGH5 was 65892.97 Da and the protein was purified to homogeneity.


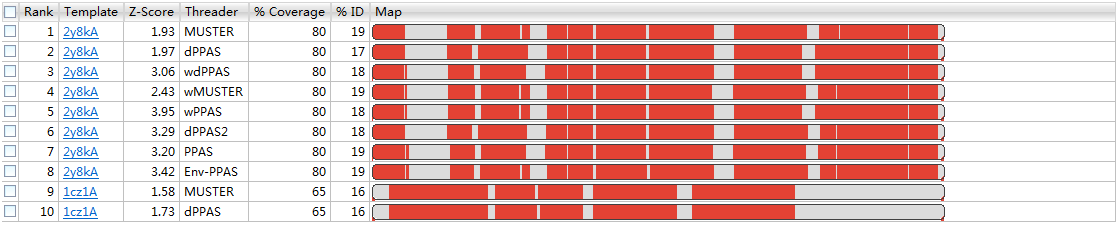
**Fig. S4.** Templates used by NovaFold for threading the structural model of CbGH5. The mapped and threaded sections were highlighted in red.


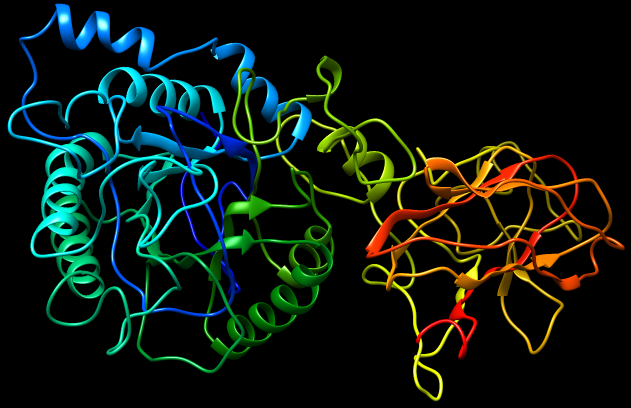

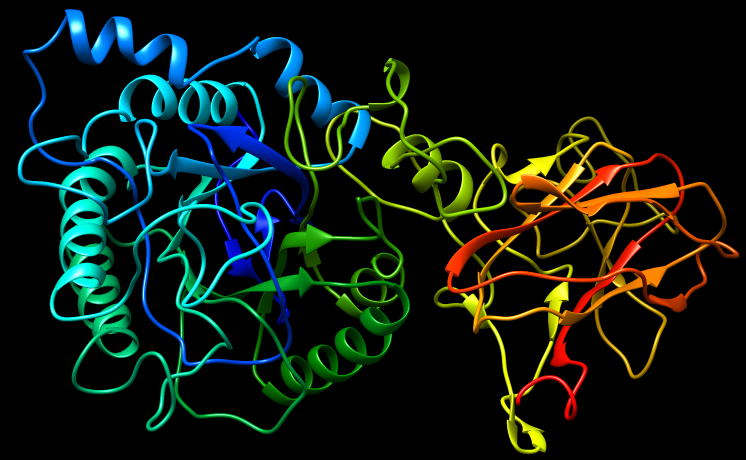
**A B**

**Fig. S5.** Comparison of the structural models of the CbGH5 wild-type **(A)** and the CbGH5(I107G, L109G, L153G, L155G, L157G, F257G, F280G, F282G) multiple-site mutant **(B)**, showing that the overall skeletons are similar. Both structural models were predicted by NovaFold as described in the **Experimental procedures** section.


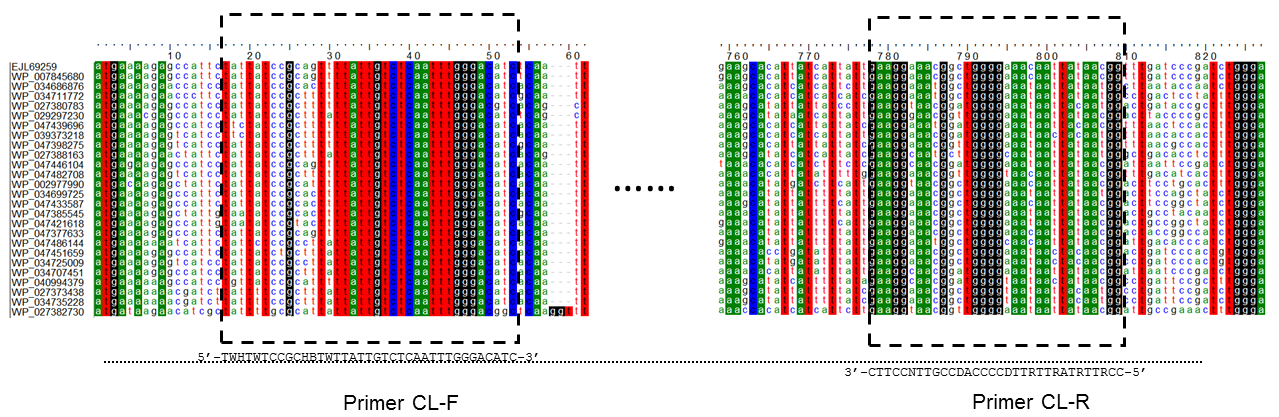


**Fig. S6.** Degenerate primers CL-F and CL-R for amplifying the core region of putative cellulase gene. Designed based on conserved sequences presented in the alignment of 27 putative cellulase genes of *Chryseobacterium* microbes.


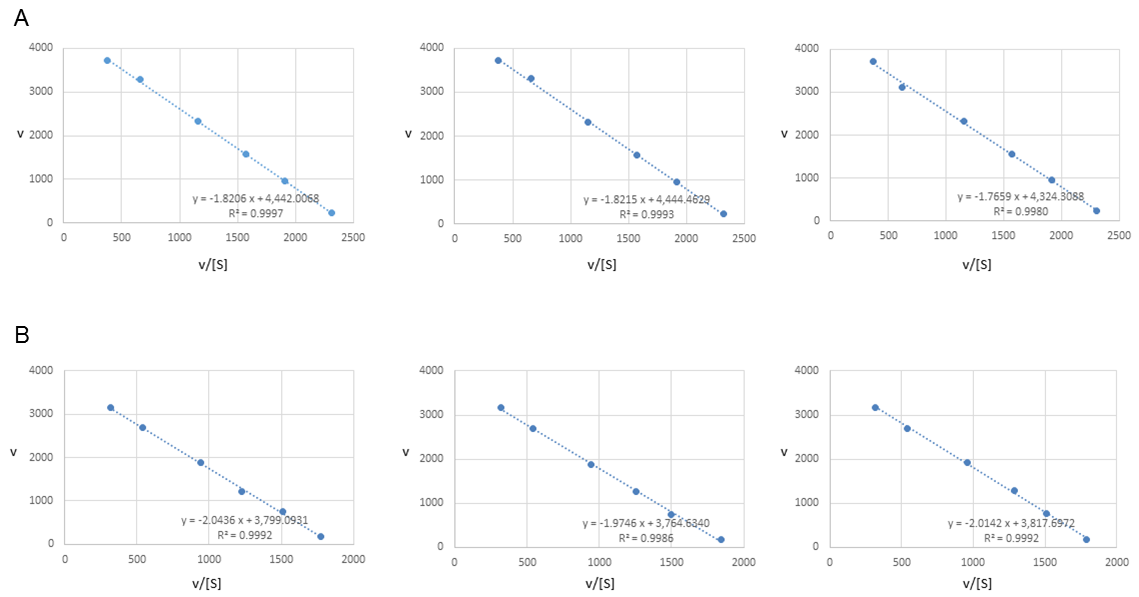


**Fig. S7.** Eadie-Hofstee plot (three replicates) to determine the *K_m_* and *V_max_* of the EG-CMC **(A)** and XYN-bw **(B)** activities of CbGH5.
